# Synchronization and resilience in the Kuramoto white matter network model with adaptive state-dependent delays

**DOI:** 10.1186/s13408-020-00091-y

**Published:** 2020-09-16

**Authors:** Seong Hyun Park, Jérémie Lefebvre

**Affiliations:** 1 University of Toronto, St. George, 40 St. George St., M5S 2E4 Toronto, Canada; 2 Krembil Research Institute, University Health Network, 60 Leonard Avenue, M5T 2S8 Toronto, Canada; 3 University of Ottawa, Gendron Hall, 30 Marie Curie, K1N 6N5 Ottawa, Canada

**Keywords:** White matter plasticity, Kuramoto model, Synchronization, Delay differential equations

## Abstract

White matter pathways form a complex network of myelinated axons that regulate signal transmission in the nervous system and play a key role in behaviour and cognition. Recent evidence reveals that white matter networks are adaptive and that myelin remodels itself in an activity-dependent way, during both developmental stages and later on through behaviour and learning. As a result, axonal conduction delays continuously adjust in order to regulate the timing of neural signals propagating between different brain areas. This delay plasticity mechanism has yet to be integrated in computational neural models, where conduction delays are oftentimes constant or simply ignored. As a first approach to adaptive white matter remodeling, we modified the canonical Kuramoto model by enabling all connections with adaptive, phase-dependent delays. We analyzed the equilibria and stability of this system, and applied our results to two-oscillator and large-dimensional networks. Our joint mathematical and numerical analysis demonstrates that plastic delays act as a stabilizing mechanism promoting the network’s ability to maintain synchronous activity. Our work also shows that global synchronization is more resilient to perturbations and injury towards network architecture. Our results provide key insights about the analysis and potential significance of activity-dependent myelination in large-scale brain synchrony.

## Introduction

Synchronization, the mechanism by which oscillatory processes collectively organize to align their phase, has been the focus of intense research across the field of non-linear dynamics [1], especially in the biological sciences due to its involvement in myriads of physiological processes. In the brain, such synchronized oscillatory patterns, observed in the rhythmic discharge of neuronal electrical impulses, play a central role in neural communication, information processing, and the implementation of higher cognitive function [2]. However, the mechanisms by which these oscillations emerge and interact within and across brain microcircuits and systems remain poorly understood.

In the mammalian brain, local synchronized oscillatory activity are coordinated through a vast network of myelinated axonal fibers called white matter. The intricate white matter structure is formed under a population of glial cells called oligodendrocytes. The oligodendrocytes produce an insulating myelin sheath coiling around axonal membranes to greatly increase the conduction velocities of propagating signals between neurons. As white matter develops to adopt a genetically programmed structure, oligodendrocytes determine to which extent specific axons are myelinated. The resulting myelin structure of the network is responsible for the emergence and evolution of a rich repertoire of spatiotemporal activity patterns, notably oscillations with various spectral properties [3].

While white matter is highly relevant in shaping brain network dynamics, the mechanisms governing myelin development and its relationship with neural activity remain mostly unknown [4]. Nevertheless, the general hypothesis surrounding this topic has been shifting away from traditional viewpoints. Recent studies suggest that white matter structure undergoes continuous changes past the stages of developmental myelination and well into adulthood, rather than remaining static as initially presumed [5]. Indeed, substantial myelin formation continues to occur within the fully mature central nervous system in an activity-dependent manner [6, 7]. Furthermore, newly discovered evidence implies that white matter structure is responsive to experiences such as learning and social interactions [8]. These findings unveil potential ways white matter can restructure itself to facilitate neurological function over time. In particular, white matter characterized as a plastic, adaptive structure, can be critical in maintaining the essential oscillatory and synchronous processes found in many neural systems [9].

Despite the complexity of the neurophysiological processes involved, it has been hypothesized that myelin remodeling collectively reinforces synchronous dynamics and oscillatory coordination in large-scale brain networks [8]. From this perspective, the temporal structure of these plastic changes can provide a higher level of corrective adjustments, improving the network’s ability to converge towards phase synchrony. As a first approach to this intricate problem, we wish to use a simplified model to determine whether activity-dependent delays impact the phase coordination of coupled oscillators when compared to static delays. We have used the Kuramoto model, a phenomenological model which has a long history of applications in neuroscience, notably to study the collective organization of oscillatory neural systems [10]. The Kuramoto model, with or without time delay, has been extensively studied [1, 11, 12], and is increasingly used to model local oscillatory neural activity in brain-scale simulations informed by anatomical white matter connectivity [13–15]. Appealing for its relative simplicity, we have enabled the Kuramoto model with phase-dependent delays to examine the collective impact of adaptive conduction on coupled phase oscillators. Specifically, we performed the stability analysis of this modified system and examined its response to structural perturbations. While preliminary, these results can provide new insights into the large-scale impact of white matter remodeling and its potential role in neural synchrony.

This paper is structured as follows. In Sect. 2, we first introduce our network model composed as a system of coupled phase-oscillators with state-dependent delays. In Sect. 3, we derive the equations for an *N*-oscillator network’s synchronous state and its respective stability criteria. Section 4 applies the derived equations from Sect. 3 to a reduced two-oscillator network. Section 5 applies the derived equations from Sect. 3 to a large-dimensional oscillator network through taking an *N*-limit approximation by handling the asymptotic phases statistically. In Sect. 6, we conduct numerical experiments and provide evidence supporting the analysis in Sects. 4 and 5 through simulated results. Section 7 explores the stabilization property in the context of spontaneous changes in network connectivity and compares the resilience of the synchronized state with and without plastic delays.

## Model

We consider a prototypical model involving a network of *N* weakly-coupled non-linear and delayed Kuramoto oscillators [9] whose phases $\theta _{i}(t)$ evolve according to the following system of differential equations:

1 $$ \frac{d}{dt}\theta _{i}(t) = \omega _{i} + \frac{g}{N} \sum_{j=1}^{N} a_{ij} \sin \bigl(\theta _{j} \bigl(t - \tau _{ij}(t) \bigr) - \theta _{i}(t) \bigr),\quad 1 \leq i \leq N, $$

where $\omega _{i} \in \mathbb{R}$ is the natural frequency of oscillator *i*, and $g > 0$ is a constant global coupling gain. The coefficients $a_{ij}$ represent synaptic weights: $a_{ij} = 1$ if there is a white matter connection between oscillator *j* to *i*; otherwise, $a_{ij} = 0$. The axonal conduction delays $\tau _{ij}$ correspond to the conduction time taken by an action potential along a myelinated fiber. The propagation speed $c_{ij} = c_{ij}(t, \ell )$ fluctuates with respect to a propagating signal’s position *ℓ* along the length of an axon as saltatory conduction occurs at successive nodes of Ranvier. The conduction velocity $c_{ij}(t, \ell )$ also changes in time. As such, each conduction delay may be written as

2 $$ \tau _{ij}(t) = \int _{P_{ij}} c_{ij}^{-1}(t, \ell ) \,d \ell ,\quad 1 \leq i,j \leq N. $$

That is, a line integral along the axonal pathway $P_{ij}$ connecting oscillator *j* to *i*. It is uncertain how neural activity and/or oscillatory brain dynamics influences myelin formation. However, it is known that myelination correlates with information transmission within and across brain areas [5], which are known to involve long range synchronization [16]. To represent this in our model, we echo previous work on oscillatory neural communication [8, 9, 17] and make axonal conduction delays phase-dependent. Specifically, we assume that each conduction delay $\tau _{ij}(t)$ evolves under the following sublinear plasticity equation:

3 $$ \alpha _{\tau }^{-1} \frac{d}{dt} \tau _{ij}(t) = H\bigl(\tau _{ij}(t)\bigr) \cdot \bigl[ - \bigl(\tau _{ij}(t) - \tau _{ij}^{0}\bigr) + \kappa \cdot \sin \bigl( \theta _{j}(t) - \theta _{i}(t) \bigr) \bigr], $$

where $1 \leq i$, $j \leq N$. Here, $\alpha _{\tau }$ is the homeostatic rate constant that sets the time scale of the evolving delays. The plasticity coefficient $\kappa > 0$ sets the gain of the conduction delay changes. The initial condition for delays $\tau _{ij}(t)$ at $t = 0$ is provided by baseline lags $\tau _{ij}^{0} \geq 0$. That is, $\tau _{ij}(0) = \tau _{ij}^{0}$ for all *i*, *j*. The Heaviside function $H(\tau )$ is defined as a smooth function such that $H(\tau ) = 0$ if $\tau < 0$ and $H(\tau ) = 1$ if $\tau > \varepsilon $ for some small $\varepsilon > 0$. The Heaviside function $H(\tau )$ is present to preserve non-negativity of the delays $\tau _{ij}(t)$ at all times $t \geq 0$. For details on the construction of the smooth Heaviside function, we refer the reader to Appendix A. According to this rule, fluctuations in conduction delays are governed by an interplay between a local drift component that represents metabolic inertia towards an initial baseline lag $\tau _{ij}^{0}$ representing minimal myelination, and a forcing term that depends on the phase difference $\Delta _{ij} := \theta _{j} - \theta _{i}$ between oscillators *i* and *j*.

**Figure 1:**
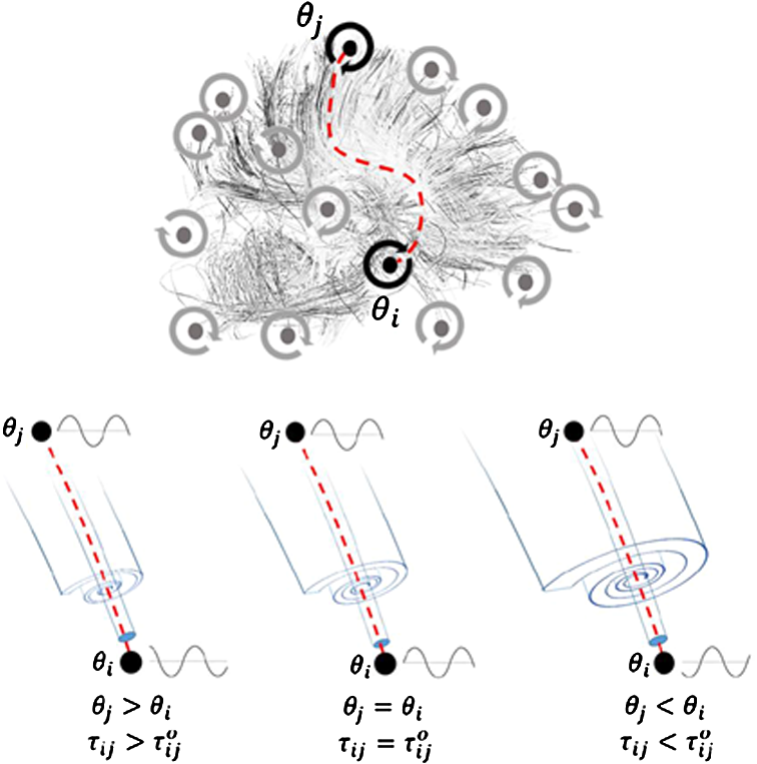
*Schematic of a network of coupled oscillators with plastic conduction delays implementing temporal control of global synchronization*. The network is built of recurrently connected phase oscillators, and connections are subjected to conduction delays. Those delays are regulated by a phase-dependent plasticity rule. Depending on whether the phase difference between two-oscillators is positive, zero, or negative, the delays are either increased (myelin retraction), unaltered, or decreased (stabilization)

A schematic of the network structure as well as the activity-dependent plasticity rule are plotted in Fig. 1. There, given $|\theta _{j} - \theta _{i}| < \pi $, one can see that whenever $\theta _{j} > \theta _{i}$, the time delay increases due to an effective reduction in conduction velocity caused by myelin retraction. The opposite occurs if $\theta _{j} < \theta _{i}$ and the time delay decreases: the connection speeds up due to myelin stabilization [8]. If the phase is the same, no changes in conduction velocity are required, and the delay remains stable at its initial lag $\tau _{ij}^{0}$.

## Synchronized state and stability with plastic delays

We are interested in characterizing the influence of the delay plasticity rule Eq. (3) towards the stability of global phase-locked solutions and, more generally, in stabilizing synchrony between neural populations (i.e. oscillators). Enabled with adaptive delays, our model corresponds to an $N + N^{2}$-dimensional functional differential equation with state-dependent delays. The analysis of such systems is technically challenging, especially in terms of stability where a modified approach must be used [18–20]. Mathematically, we analyze the network’s ability to asymptotically achieve the following synchronized state:

4 $$ \theta _{i}(t) = \varOmega t + \phi _{i} $$

for all $i \leq N$ as $t \rightarrow \infty $, where *Ω* is the global fixed frequency of the oscillators and $\phi _{i}$ is the asymptotic phase-offset of oscillator *i* as time $t \rightarrow \infty $. As we will see, adaptive delays following the plasticity rule equation (3) require non-zero phase differences $\Delta _{ij}(t) := \theta _{j}(t) - \theta _{i}(t)$ in order to maintain its equilibrium values, from which the network can no longer become in-phase. Nevertheless, the oscillators are able to become entrained to a common frequency *Ω*. Hence, we say that our network becomes synchronized when the nodes $\theta _{i}(t)$ globally oscillate at some stable frequency *Ω* and become phase-locked under individual offsets $\phi _{i}$. As we are primarily concerned with the effects of delays changes under the plastic Eq. (3), we assume that all oscillators share the same natural frequency $\omega _{i} = \omega _{0}$ for all *i* in Eq. (1). To simplify the convergent behaviour of delays $\tau _{ij}(t)$ following Eq. (3), we set $\alpha _{\tau }= 1$.

Applying the ansatz Eq. (4) onto both the phases in Eq. (1) and the delays in Eq. (3), we obtain the following expressions for the global frequency $\varOmega \in \mathbb {R}$ and individual offsets $\boldsymbol {\phi }= (\phi _{1}, \ldots , \phi _{N}) \in \mathbb {R}^{N}$:

5 $$\begin{aligned}& \varOmega = \omega _{0} + \frac{g}{N} \sum _{j=1}^{N} a_{ij} \sin \bigl(- \varOmega \tau _{ij}^{E} + \Delta _{ij}\bigr), \end{aligned}$$

6 $$\begin{aligned}& \tau _{ij}^{E} = \max \bigl(\tau _{ij}^{0} + \kappa \sin (\Delta _{ij}), 0\bigr), \end{aligned}$$

for all *i*, *j*, where $\Delta _{ij} = \phi _{j} - \phi _{i}$. It must be true that if *Ω* satisfies Eq. (5), then $\varOmega \in [\omega _{0} - g, \omega _{0} + g]$. To assess the network’s stability at the synchronized state $(\varOmega , \boldsymbol {\phi })$ satisfying Eqs. (5) and (6), we introduce the perturbation terms $\epsilon _{i}(t)$, $\eta _{ij}(t)$ around the equilibrium states Eq. (4) and Eq. (6) for $\theta _{i}(t)$ and $\tau _{ij}(t)$, respectively. That is, we write

7 $$\begin{aligned}& \theta _{i}(t) = \varOmega t + \phi _{i} + \epsilon _{i}(t), \end{aligned}$$

8 $$\begin{aligned}& \tau _{ij}(t) = \tau _{ij}^{E} + \eta _{ij}(t), \end{aligned}$$

and examine the stability at the equilibrium point $\epsilon _{i}(t) = \eta _{ij}(t) = 0$. The delay perturbations $\eta _{ij}(t)$ abide by the linearized form of Eq. (3) around all positive delays $\tau _{ij}^{E} = \tau _{ij}^{0} + \kappa \sin (\Delta _{ij}) > 0$. For the equilibrium delays $\tau _{ij}^{E} = 0$, we proceed by assuming the corresponding perturbation terms will act in an asymptotically stable manner. Specifically, for all phase-offset differences such that $\tau _{ij}^{E} = 0$, by the nature of the Heaviside cutoff function $H(\tau )$ in Eq. (3) there exists some $t_{\mathrm{asy}} \in \mathbb {R}$ such that for all $t > t_{\mathrm{asy}}$, $\tau _{ij}(t) = 0$ and consequently $\eta _{ij}(t) = 0$. That is, if $\tau _{ij}^{E} = 0$, the perturbation term is asymptotically $\eta _{ij}(t) = 0$. This condition will be satisfied given the synchronous state $(\varOmega , \boldsymbol {\phi })$ is indeed stable. Since the purpose of linearization is to determine stability, the above assumption is valid to make before proceeding. Taken together, the linearized equations for the terms $\eta _{ij}(t)$ as $\tau _{ij}(t) \rightarrow \tau _{ij}^{E}$ are given by

9 $$ \frac{d}{dt} \eta _{ij}(t) = \textstyle\begin{cases} -\eta _{ij}(t) + \kappa C_{ij}^{0} (\epsilon _{j}(t) - \epsilon _{i}(t)) & \text{if } \tau _{ij}^{E} > 0, \\ 0 & \text{if } \tau _{ij}^{E} = 0, \end{cases} $$

where $C_{ij}^{0} := \cos (\Delta _{ij})$. We can use the linearization approach in the context of equations with state-dependent delays [18–20] as follows. Inserting Eq. (7) and Eq. (8) into the Kuramoto equation (1),

10 $$ \frac{d}{dt}\epsilon _{i}(t) = \omega _{0} - \varOmega + \frac{g}{N} \sum _{j=1}^{N} a_{ij}\sin \bigl( -\varOmega \bigl(\tau _{ij}^{E} + H\bigl(\tau _{ij}^{E} \bigr)\eta _{ij}(t)\bigr) + \Delta _{ij} + \epsilon _{j}\bigl(t-\tau _{ij}^{E}\bigr) - \epsilon _{i}(t) \bigr), $$

where we have made the approximation $\epsilon _{j}(t - \tau _{ij}(t)) \approx \epsilon _{j}(t - \tau _{ij}^{E})$ when $\tau _{ij}(t)$ is near $\tau _{ij}^{E}$. With $(\varOmega , \boldsymbol {\phi })$ satisfying Eq. (5), and by taking a first-order expansion around the term $-\varOmega \tau _{ij}^{E}$, we obtain the linear system for the phase perturbation term given by

11 $$ \frac{d}{dt}\epsilon _{i}(t) = \frac{g}{N}\sum_{j=1}^{N} a_{ij}C_{ij}\bigl[ \epsilon _{j}\bigl(t - \tau _{ij}^{E}\bigr) - \epsilon _{i}(t) - \varOmega H_{ij}\eta _{ij}(t)\bigr], $$

where we denote $C_{ij} = \cos (-\varOmega \tau _{ij}^{E} + \Delta _{ij})$ and $H_{ij} = H(\tau _{ij}^{E})$. Hence, the network synchronizes at $(\varOmega , \boldsymbol {\phi })$ given that all perturbation terms $\epsilon _{i}(t)$, $\eta _{ij}(t)$ following the linear system of fixed delay differential Eqs. (9) and (11) converges to 0. From here, we can analyze the stability at the synchronous point $(\varOmega , \boldsymbol {\phi })$ by setting the ansatz $\epsilon _{i}(t) = v_{i}e^{\lambda t}$, $\eta _{ij}(t) = w_{ij} e^{ \lambda t}$ with respect to eigenvalue $\lambda \in \mathbb{C}$. By Eq. (9), the coefficients $w_{ij}$ satisfy

12 $$ w_{ij} = \kappa C_{ij}^{0} H_{ij} \biggl( \frac{v_{j} - v_{i}}{\lambda + 1} \biggr) $$

for all *i*, *j*, $\lambda \neq -1$. Applying Eq. (12) to the coefficients $w_{ij}$,

13 $$ \lambda v_{i} = \frac{g}{N} \sum _{j=1}^{N} a_{ij} C_{ij} \biggl[ v_{j} e^{-\lambda \tau _{ij}^{E}} - v_{i} - \varOmega \kappa C_{ij}^{0} H_{ij} \biggl( \frac{v_{j} - v_{i}}{\lambda + 1} \biggr) \biggr] .$$

That is, *λ* is an eigenvalue if there exists an eigenvector $\vec{v} = (v_{1}, \ldots , v_{N}) \in \mathbb {C}^{N}$ satisfying Eq. (13). In matrix form, Eq. (13) can be expressed as $M_{\lambda }\vec{v} = \vec{0}$, where $M_{\lambda }= (M_{ij})$ is the *N*-dimensional square matrix with entries

14 $$\begin{aligned} \begin{aligned} &M_{ii} = \lambda (\lambda + 1) + \frac{g}{N} \sum_{j=1}^{N} a_{ij}C_{ij}\bigl( \lambda + 1 - \varOmega \kappa C_{ij}^{0} H_{ij}\bigr), \\ &M_{ij} = \frac{g}{N}a_{ij}C_{ij} \bigl[\varOmega \kappa C_{ij}^{0}H_{ij} - (\lambda +1) e^{-\lambda \tau _{ij}^{E}} \bigr],\quad i \neq j. \end{aligned} \end{aligned}$$

In summary, we acquire the following stability criterion: The system is stable around the synchronized state $(\varOmega , \boldsymbol {\phi })$ if for all eigenvalues $\lambda \in \mathbb{C}$ satisfying $\det M_{\lambda }= 0$, $\operatorname {Re}(\lambda ) < 0$. Appendix A discusses the existence and uniqueness of solutions $\theta _{i}(t)$, $\tau _{ij}(t)$, as well as the justification of the above linearization and respective stability analysis.

Of note is that our approach is an extension of the non-plastic case. Indeed, without plasticity $\kappa = 0$, the delays remain fixed at the baseline lag $\tau _{ij}^{0}$, and as a result there is no need for the delays to establish an equilibrium with positive phase-offset differences $\Delta _{ij}$. Hence, the oscillators become perfectly in-phase with $\phi _{i} = 0$ during synchronization. Consequently, Eq. (5) for the global frequency *Ω* reduces to the one-dimensional fixed-point expression

15 $$ \varOmega = \omega _{0} + \frac{g}{N}\sum _{j=1}^{N} a_{ij} \sin \bigl(- \varOmega \tau _{ij}^{0}\bigr). $$

By employing similar steps as above, the phase perturbation terms $\epsilon _{i}(t)$ follow the linear system

16 $$ \frac{d}{dt}\epsilon _{i}(t) = \frac{g}{N}\sum_{j=1}^{N} a_{ij} \cos \bigl(- \varOmega \tau _{ij}^{0} \bigr) \bigl(\epsilon _{j}\bigl(t - \tau _{ij}^{0} \bigr) - \epsilon _{i}(t)\bigr) $$

resulting in the corresponding eigenvalue matrix $M_{\lambda }$ defined with entries

17 $$\begin{aligned} \begin{aligned} &M_{ii} = \lambda + \frac{g}{N}\sum_{j=1}^{N} a_{ij} \cos \bigl(-\varOmega \tau _{ij}^{0} \bigr), \\ &M_{ij} = -\frac{g}{N}a_{ij} \cos \bigl(- \varOmega \tau _{ij}^{0}\bigr) e^{- \lambda \tau _{ij}^{0}}. \end{aligned} \end{aligned}$$

With non-plastic delays, stability analysis at $\theta _{i}(t) = \varOmega t$ has been accomplished through other means. Lyapunov functionals [21] have shown that a sufficient criterion for synchronization around $\theta _{i}(t) = \varOmega t$ is $\cos (\varOmega \tau _{ij}^{0}) > 0$ for all active connections *i*, *j* such that $a_{ij} =1$, and *Ω* satisfies Eq. (15). This criterion becomes necessary given a unique baseline lag $\tau _{ij}^{0} = \tau ^{0}$ [22]. That is, it can be shown that the oscillators synchronize if and only if $\cos (\varOmega \tau ^{0}) > 0$.

## Synchronization of two coupled oscillators with plastic delays

As a first illustrative approach to the general case when the number of coupled oscillators is large, let us first consider a reduced two-oscillator network. The following setup is similar to the system analyzed in [23]. We first let $N = 2$ with $a_{12} = a_{21} = 1$ and remove all self-integration terms by setting $a_{11} = a_{22} = 0$. We also set the baseline delays to be equal with $\tau _{12}^{0} = \tau _{21}^{0} = \tau ^{0}$. Then the phases $\theta _{1}(t)$, $\theta _{2}(t)$ follow the Kuramoto system,

18 $$\begin{aligned} \begin{aligned} &\theta _{1}'(t) = \omega _{0} + g \sin \bigl(\theta _{2}\bigl(t - \tau _{12}(t)\bigr) - \theta _{1}(t)\bigr), \\ &\theta _{2}'(t) = \omega _{0} + g \sin \bigl(\theta _{1}\bigl(t - \tau _{21}(t)\bigr) - \theta _{2}(t)\bigr), \end{aligned} \end{aligned}$$

with respective plasticity equations

19 $$ \begin{aligned} &\tau _{12}'(t) = H\bigl(\tau _{12}(t)\bigr) \cdot \bigl[-\bigl(\tau _{12}(t) - \tau ^{0}\bigr) + \kappa \sin \bigl(\theta _{2}(t) - \theta _{1}(t)\bigr)\bigr], \\ &\tau _{21}'(t) = H\bigl(\tau _{21}(t) \bigr) \cdot \bigl[-\bigl(\tau _{21}(t) - \tau ^{0}\bigr) - \kappa \sin \bigl(\theta _{2}(t) - \theta _{1}(t) \bigr)\bigr]. \end{aligned} $$

By symmetry we may let $\Delta _{12} = \phi _{2} - \phi _{1} > 0$. We proceed by setting a sufficiently large plasticity gain with $\kappa \gg \tau ^{0}$. Then it follows that the respective equilibrium delays are $\tau _{12}^{E} := \tau ^{0} + \kappa \sin (\Delta _{12})$ and $\tau _{21}^{E} = 0$, resulting in a single positive equilibrium delay. Hence, by Eq. (5) the frequency *Ω* and offset $\Delta _{12}$ satisfies

20 $$\begin{aligned}& \varOmega = \omega _{0} + g \sin \bigl(-\varOmega \tau _{12}^{E} + \Delta _{12}\bigr), \end{aligned}$$

21 $$\begin{aligned}& \varOmega = \omega _{0} - g \sin (\Delta _{12}). \end{aligned}$$

**Figure 2:**
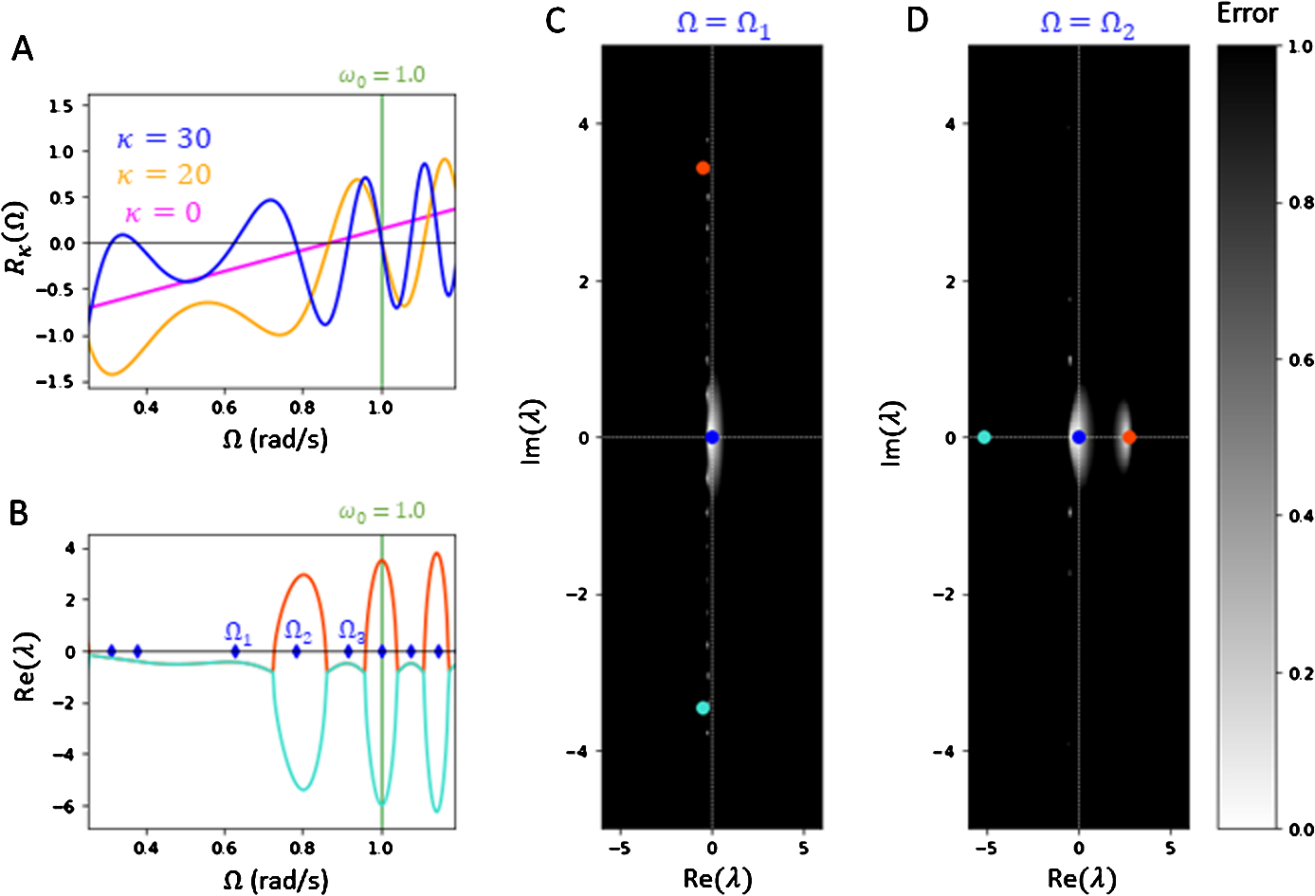
*Theoretical stability plots for two-oscillator system*. **A**. Plot of error functions $R_{\kappa }(\varOmega )$ with varying fixed gain $\kappa = 0$ (magenta), $\kappa = 20$ (yellow), $\kappa = 30$ (blue). All roots $\varOmega \in [\omega _{0} - g, \omega _{0})$ of $R_{\kappa }(\varOmega )$ are potential synchronization frequencies for the two-oscillator system. The number of roots *Ω* for $\mathcal{R}_{\kappa }(\varOmega ) = 0$ increase with larger *κ*. **B**. The plasticity gain is set to $\kappa = 30$. Plot of the real part of the non-zero branches $\lambda _{1}(\varOmega )$, $\lambda _{2}(\varOmega )$ (orange, cyan) of the polynomial root equation $P_{\varOmega }(\lambda ) + Q_{\varOmega }(\lambda ) = 0$ over $\varOmega \in [\omega _{0} - g, \omega _{0})$. Ticks on the *Ω*-axis (blue) indicate the frequencies $\varOmega _{i}$ solving $\mathcal{R}_{\kappa }(\varOmega _{i}) = 0$ where the system can synchronize. The plotted branches imply that the oscillators will synchronize at $\varOmega = \varOmega _{1}, \varOmega _{3}$, and avoid the unstable frequency $\varOmega = \varOmega _{2}$ with $\operatorname {Re}\lambda _{1}(\varOmega _{2}) > 0$. **C**, **D**. Error heatmaps with $\varOmega = \varOmega _{1}, \varOmega _{2}$, respectively, approximate the distribution of eigenvalues $\lambda \in \mathbb {C}$ solving $P_{\varOmega }(\lambda ) + Q_{\varOmega }(\lambda )e^{-\lambda \tau } = 0$ near $\lambda = 0$, scaled and normalized for visibility. Spots near zero error (white) suggest potential eigenvalue locations. Markers plot the eigenvalues $\lambda _{0} = 0, \lambda _{1}(\varOmega ), \lambda _{2}(\varOmega )$ (blue, orange, cyan) for $\tau = 0$. The heatmap in **D** indicates an eigenvalue *λ* near $\lambda _{1}(\varOmega _{2}) > 0$, which implies instability at $\varOmega = \varOmega _{2}$. All other eigenvalues *λ* appear to be distributed either at $\lambda = 0$ or on the left-side of the imaginary axis. Here, $\varOmega _{1} = 0.626$ and $\varOmega _{2} = 0.783$. For all plots, $\alpha _{\tau }= 0.5$, $g = 1.5/2$, $\omega _{0} = 1.0$, $\kappa = 30$, and $\tau ^{0} = 0.1~\text{s}$

Solving for $\Delta _{12}$ above, we obtain

22 $$ \Delta _{12} = \arcsin \biggl( \frac{\omega _{0} - \varOmega }{g} \biggr) > 0, $$

where positivity holds only when $\varOmega < \omega _{0}$. This leads to the oscillators synchronizing at a lower frequency $\varOmega \in [\omega _{0} - g, \omega _{0})$. Substituting $\Delta _{12}$ in Eq. (20) with Eq. (22), if we define the root function

23 $$ \mathcal{R}_{\kappa }(\varOmega ) := \varOmega -\omega _{0} - g \sin \biggl(- \varOmega \biggl(\tau ^{0} + \kappa \biggl( \frac{\omega _{0} - \varOmega }{g} \biggr) \biggr) + \arcsin \biggl( \frac{\omega _{0} - \varOmega }{g} \biggr) \biggr) , $$

then $\theta _{1}(t)$, $\theta _{2}(t)$ synchronizes at a common frequency *Ω* implicitly satisfying $R_{\kappa }(\varOmega ) = 0$. Since it was assumed that $\tau _{21}^{E} = 0$, for self-consistency the synchronous frequency *Ω* must also satisfy $\tau ^{0} - \kappa \sin (\Delta _{12}) < 0$, which can be written as $\varOmega < \omega _{0} - g\kappa ^{-1}\tau ^{0} \approx \omega _{0}$ since we set $\kappa \gg \tau ^{0}$. Without plasticity $\kappa = 0$, the oscillators become in-phase with $\Delta _{12} = 0$ and synchronizes at a frequency *Ω* that is a root of the function

24 $$ \mathcal{R}_{0}(\varOmega ) := \varOmega - \omega _{0} + g \sin \bigl(\varOmega \tau ^{0}\bigr) $$

as stated by Eq. (15). Figure 2(A) plots functions $\mathcal{R}_{\kappa }(\varOmega )$ on the interval $[\omega _{0}-g, \omega _{0})$ with respect to representative values $\kappa = 0, 20, 30$ of the plasticity gain. We observe that higher plasticity gains $\kappa > 0$ generally leads to a greater number of potential synchronization frequencies *Ω* for our system. In Fig. 2(A), one can see that the functions $\mathcal{R}_{0}(\varOmega )$ and $\mathcal{R}_{20}(\varOmega )$ have single roots within the interval $[\omega _{0}-g, \omega _{0})$, while $\mathcal{R}_{30}(\varOmega )$ has five.

The stability in the two-dimensional case can be easily determined. Indeed, as derived in Sect. 3, the stability of the oscillators at the synchronization state $(\varOmega , \Delta _{12})$ is determined by the distribution of eigenvalues $\lambda \in \mathbb {C}$ that satisfy

25 $$ \det \begin{vmatrix} \lambda (\lambda + 1) + C_{12}(\lambda + 1 - \varOmega \kappa C_{0}) & -C_{12}(e^{- \lambda \tau _{12}^{E}}(\lambda + 1) - \varOmega \kappa C_{0}) \\ -C_{21} & \lambda + C_{21} \end{vmatrix} = 0, $$

where $C_{12} = g \cos (-\varOmega \tau _{12}^{E} + \Delta _{12})$, $C_{21} = g \cos (\Delta _{12})$, $C_{0} = \cos (\Delta _{12})$. This results in the root equation $P_{\varOmega }(\lambda ) + Q_{\varOmega }(\lambda )e^{-\lambda \tau } = 0$, where $\tau = \tau _{12}^{E}$ is the single positive delay and $P_{\varOmega }(\lambda )$, $Q_{\varOmega }(\lambda )$ are polynomials given by

26 $$\begin{aligned}& P_{\varOmega }(\lambda ) = \lambda ^{3} + (1+C_{12}+C_{21}) \lambda ^{2} + \bigl[ \tilde{\kappa }C_{12} + (1+C_{12})C_{21}\bigr]\lambda + C_{12}C_{21} , \end{aligned}$$

27 $$\begin{aligned}& Q_{\varOmega }(\lambda ) = -C_{12}C_{21}(\lambda +1), \end{aligned}$$

where $\tilde{\kappa } = 1 - \varOmega \kappa C_{0}$. Note that $P_{\varOmega }(0) + Q_{\varOmega }(0) = 0$, which means we have neutral stability. If we make the approximation $\tau \approx 0$, the eigenvalues *λ* correspond to exact cubic roots of $P_{\varOmega }(\lambda ) + Q_{\varOmega }(\lambda )$. It is possible for the stability of the system under $\tau > 0$ to align with the stability under $\tau = 0$, particularly for small *τ*. If we ignore the neutral stability of our zero transcendental equation, Theorem 1 of [24] states that under certain conditions, the stability of the system at $\tau = 0$ and at any $\tau > 0$ does not change. For rigorous purposes however, we are still interested in finding the distribution of eigenvalues $\lambda \in \mathbb {C}$ solving Eq. (25), particularly if it is accessible through numerical approximation.

Setting the plasticity gain $\kappa = 30$, Fig. 2(B) shows the real parts of the two non-zero branches $\lambda _{1}$, $\lambda _{2}$ of the roots $P_{\varOmega }(\lambda ) + Q_{\varOmega }(\lambda ) = 0$, plotted with respect to the global frequency *Ω*. If any of the branches are positive, it implies by the above discussion that the oscillators will not synchronize at the state $(\varOmega , \Delta _{12})$, where $\Delta _{12} = \Delta _{12}(\varOmega )$ is given by Eq. (22). We denote $\varOmega _{1} < \varOmega _{2} < \varOmega _{3}$ to be the three largest synchronization frequencies corresponding to equilibria solutions $\varOmega _{i} < \omega _{0}$ of the root equation $\mathcal{R}_{\kappa }(\varOmega _{i}) = 0$ as presented in Fig. 2(A). The eigenvalue branches imply that frequencies $\varOmega _{1}$, $\varOmega _{3}$ are stable with $\operatorname {Re}(\lambda _{1}), \operatorname {Re}(\lambda _{2}) < 0$ at $\varOmega = \varOmega _{1}, \varOmega _{3}$, while $\varOmega _{2}$ is an unstable frequency with $\operatorname {Re}(\lambda _{1}) > 0$ at $\varOmega = \varOmega _{2}$. Figure 2(C), (D) shows heatmap approximations of complex roots *λ* of Eq. (24) at $\varOmega = \varOmega _{1}$, $\varOmega = \varOmega _{2}$, respectively. We can see that at $\varOmega = \varOmega _{1}$, both non-zero cubic roots are located on the left-side of the imaginary axis, and the error heatmap shows that the eigenvalues are located on the left-hand side of the imaginary axis. At $\varOmega = \varOmega _{2}$, all cubic roots are real with a single positive root $\lambda _{1} > 0$. Consistent with the stability of our system at $\tau = 0$, the error heatmap implies there exists an eigenvalue $\lambda \in \mathbb {C}$ satisfying Eq. (25) near the positive root $\lambda _{1}$. This highlights that under sufficiently large plasticity gain, adaptive delays introduce multiple stable states $(\varOmega , \Delta _{12})$ in a two-oscillator system, whose stability can be assessed under the zero delay approximation $\tau _{12}^{E} \approx 0$. Our stability analysis reveals that the oscillators can synchronize at multiple possible frequencies, which suggests a greater degree of adaptability in our system.

## Synchronization in large-scale oscillator networks with plastic delays

Using inspiration by the two-dimensional case in Sect. 4, we consider here a large-dimensional system using *N*-limit approximations. Again for simplicity, we set the baseline lags to be constant with $\tau _{ij}^{0} \equiv \tau ^{0}$ and the connection topology to be all-to-all with $a_{ij} \equiv 1$. We approach the synchronization state $(\varOmega , \boldsymbol {\phi })$ of the network with adaptive delays, given by Eqs. (5) and (6), in the following statistical sense. Suppose that the phase-offsets $\phi _{i}$ are i.i.d. under some distribution. We can set the offsets to be centered at 0 by defining the re-centered offsets $\Delta _{i} := \phi _{i} - \overline{\phi }$, where *ϕ̅* is the mean of *ϕ*. Then $\Delta _{ij} = \phi _{j} - \phi _{i} = \Delta _{j} - \Delta _{i}$. We take $\Delta _{i}$ to be i.i.d. under some density function $\rho (\Delta )$. Setting a random $\Delta _{i}$ in the global frequency equation (5) for each $i \leq N$, and then taking the limit $N \rightarrow \infty $, we obtain the following *N*-limit approximation for frequency *Ω* and density $\rho (\Delta )$:

28 $$ \varOmega = \omega _{0} + g \int _{-\infty }^{\infty }\sin \bigl(-\varOmega \tau ^{E}\bigl( \Delta - \Delta '\bigr) + \bigl(\Delta - \Delta '\bigr)\bigr)\rho (\Delta ) \,d\Delta $$

for all fixed $\Delta ' \in \mathbb {R}$, where $\tau ^{E}(\Delta ) = H(\tau ^{0} + \kappa \sin \Delta ) \cdot (\tau ^{0} + \kappa \sin \Delta )$. In order to apply Eq. (28) to obtain the global frequencies *Ω*, we parametrize the unknown density $\rho (\Delta )$ by assuming it is Gaussian under some small phase-offset variance $\delta ^{2}$. That is, we have

29 $$ \rho _{\delta }(\Delta ) = \frac{1}{\sqrt{2\pi \delta ^{2}}} \exp \biggl( \frac{-\Delta ^{2}}{2\delta ^{2}} \biggr)$$

and we set $\rho (\Delta ) = \rho _{\delta }(\Delta )$. If the variance $\delta ^{2}$ is small, we can approximate the fixed offset in Eq. (28) as $\Delta ' \approx 0$, as well as make the approximation $\tau ^{E}(\Delta ) \approx H(\tau ^{0} + \kappa \Delta ) \cdot (\tau ^{0} + \kappa \Delta )$ since Δ is small. Hence, our large-scale network synchronizes near a global frequency *Ω* with Gaussian distributed phase-offsets with variance $\delta ^{2}$ given that $(\varOmega , \delta ^{2})$ is a root of the function

30 $$ \mathcal{R}\bigl(\varOmega , \delta ^{2}\bigr) := \omega _{0} - \varOmega + g \int _{- \infty }^{\infty }\sin \bigl(-\varOmega \tau ^{E}(\Delta ) + \Delta \bigr)\rho _{\delta }(\Delta ) \,d\Delta .$$

Without plasticity, we recall that we have in-phase synchronization at global frequency *Ω* which is a solution to $\mathcal{R}_{0}(\varOmega , 0) = 0$. That is,

31 $$ \varOmega = \omega _{0} + g\sin \bigl(-\varOmega \tau ^{0}\bigr) .$$

**Figure 3:**
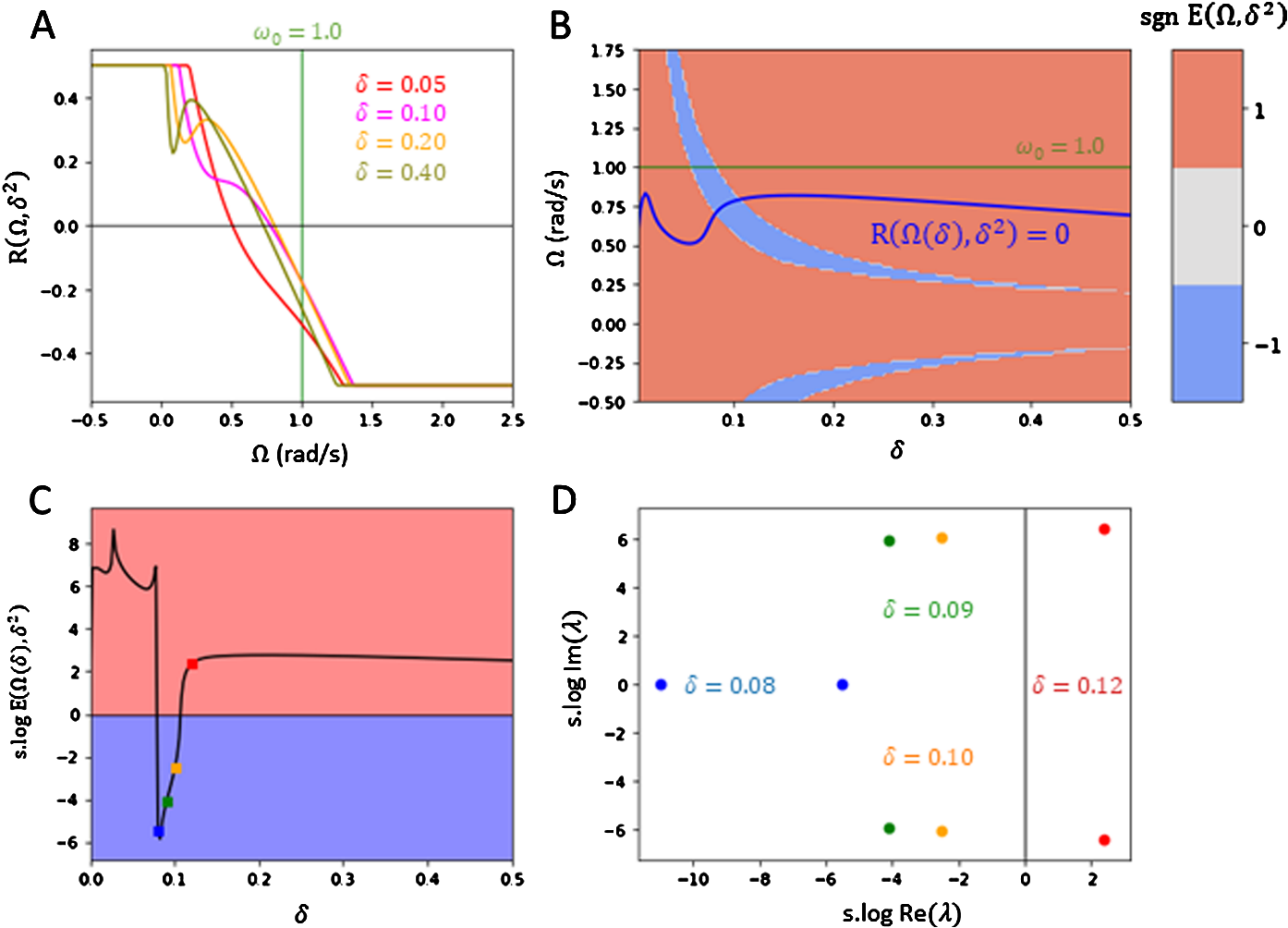
*Theoretical stability plots for large* *N-dim oscillator system*. **A**. Plots of error function $\mathcal{R}(\varOmega , \delta ^{2})$ with varying fixed $\delta > 0$ over $\varOmega \in [\omega _{0}-g, \omega _{0}+g]$. The function is truncated between interval $[-0.5, 0.5]$ for visibility. There is a unique root $\mathcal{R}(\varOmega , \delta ^{2}) = 0$ for each fixed $\delta > 0$. **B**. Colour map of $\operatorname {sgn}E(\varOmega , \delta ^{2})$ over states $(\varOmega , \delta ^{2}) \in [\omega _{0}-g, \omega _{0}+g] \times (0, 0.5^{2})$, along with the implicit solution curve (purple) $\varOmega = \varOmega (\delta )$ parametrizing level set $\mathcal{R}(\varOmega , \delta ^{2}) = 0$. Stable regions correspond to $\operatorname {sgn}E(\varOmega , \delta ^{2}) = -1$ (blue) and unstable regions correspond to $\operatorname {sgn}E(\varOmega , \delta ^{2}) = 1$ (red). The network synchronizes near a state $(\varOmega (\delta ), \delta ^{2})$ overlapping the stable region. **C**. Plot of stability term $\text{s.} \log E(\varOmega , \delta ^{2})$ along the solution curve $\varOmega = \varOmega (\delta )$ over $\delta \in (0, 0.5)$. There is a small interval $\delta \in (0.08, 0.1)$ for which $(\varOmega (\delta ), \delta ^{2})$ is in the stable region (blue). Other states are in the unstable region (red). **D**. Complex plot of non-zero eigenvalues of $P(\lambda \mid \varOmega , \delta ^{2}) + Q(\lambda \mid \varOmega , \delta ^{2})$ on solution states $(\varOmega (\delta ^{2}), \delta ^{2})$ across varying $\delta > 0$, scaled by s.log for visibility. The eigenvalues in plot **D** were computed at respective states $(\varOmega , \delta ^{2})$ in plot **C** indicated by the same colour. Power terms for polynomial $Q(\lambda \mid \varOmega , \delta ^{2})$ were computed up to degree $M = 3$. The parameters used for all plots are $\alpha _{\tau }= 1.0$, $g = 1.5$, $\omega _{0} = 1.0$, $\kappa = 80$, and $\tau ^{0} = 0.1~\text{s}$

Figure 3(A) plots the curve $\mathcal{R}(\varOmega , \delta ^{2})$ for various fixed-values of $\delta > 0$ on $\varOmega \in [\omega _{0} - g, \omega _{0} + g]$. We find that there is a unique but different root *Ω* to $\mathcal{R}(\varOmega , \delta ^{2}) = 0$ at each fixed variance $\delta ^{2} > 0$. Hence, we can obtain an implicit curve $\varOmega = \varOmega (\delta )$ by parametrizing the level curve $\mathcal{R}(\varOmega , \delta ^{2}) = 0$ with respect to $\delta > 0$. The curve is plotted in Fig. 3(B), and shows a continuous range of potential synchronization states $(\varOmega (\delta ), \delta ^{2})$ along $\delta > 0$ to a large *N*-dimensional system of oscillators. The graph of the level curve $\mathcal{R}(\varOmega , \delta ^{2}) = 0$ also shows that $\varOmega (\delta ) < \omega _{0}$ for all $\delta > 0$, implying that the oscillators are drawn to synchronize at a lower frequency from their natural frequency.

For small offset differences, the equilibrium delays are approximately $\tau _{ij}^{E} \approx \tau ^{0} + \kappa \Delta _{ij}$ if $\Delta _{ij} > 0$, and $\tau _{ij}^{E} = 0$ if $\Delta _{ij} < 0$. Before proceeding, we set *κ* to be sufficiently larger than the baseline lag $\tau ^{0}$ such that $\frac{\tau ^{0}}{\kappa } \approx 0$. Then most negative offset differences $\Delta _{ij} < 0$ fall below the Heaviside cutoff $\tau ^{0} + \kappa \Delta _{ij} < 0$. From this, the Heaviside term becomes approximately dependent to the sign of $\Delta _{ij}$ with $H_{ij} \approx H(\Delta _{ij})$ for all *i*, *j*. For large gain *κ*, two categories of equilibrium delays emerge that contribute to the global frequency *Ω* in Eq. (30): For half the connections with offset difference $\Delta _{ij} < 0$, the corresponding delays $\tau _{ij}(t)$ decay to $\tau _{ij}^{E} = 0$ as we have $\tau ^{0} < -\kappa \Delta _{ij} < 0$ with $\tau ^{0} \ll \kappa $. Otherwise, with $\Delta _{ij} > 0$ the delay $\tau _{ij}(t)$ establishes a positive equilibrium at $\tau _{ij}^{E} = \tau ^{0} + \kappa \Delta _{ij}$. The positive delays $\tau _{ij}^{E}$ become widely distributed under large standard deviation *κδ*.

The coupled network can synchronize towards any stable point $(\varOmega , \delta ^{2})$ along the curve $R(\varOmega , \delta ^{2}) = 0$. To assess the stability at each state $(\varOmega , \delta ^{2})$, consider our *N*-dimensional eigenvalue stability criterion $M_{\lambda }\vec{v} = 0$ for eigenvector $\vec{v} \in \mathbb {C}^{N}$ and matrix $M_{\lambda }$ whose entries are given by Eq. (13). That is,

32 $$ \begin{aligned}[b] & \Biggl[ \lambda (\lambda +1) + \frac{g}{N} \sum_{j=1}^{N} C_{\varOmega }( \Delta _{ij}) \bigl(\lambda + 1 - \tilde{\kappa }C_{0}(\varOmega )H(\Delta _{ij})\bigr) \Biggr] v_{i} \\ &\quad {} + \frac{g}{N} \sum_{j=1}^{N} C_{\varOmega }(\Delta _{ij}) \bigl( \tilde{\kappa }C_{0}(\Delta )H(\Delta _{ij}) - (\lambda +1)e^{-\lambda \tau _{ij}^{E}}\bigr)v_{j} = 0 \end{aligned} $$

for all $i \leq N$, where $\tilde{\kappa } = \varOmega \kappa $, $C_{0}(\Delta ) = \cos (\Delta )$, $C_{\varOmega }(\Delta ) = \cos (-\varOmega \tau ^{E}(\Delta ) + \Delta )$, and $\tau ^{E}(\Delta ) = H(\Delta )(\tau ^{0} + \kappa \Delta )$. Once again, if our offset variance $\delta ^{2}$ is small for Eq. (31) we can approximate the terms $\Delta _{ij} = \Delta _{j} - \Delta _{i} \approx \Delta _{j}$ by assuming $\Delta _{i} \approx 0$ at each *i*. We derive an *N*-limit version of the eigenvalue Eq. (32) as follows. Likewise to the frequency equation *Ω*, we can obtain a similar *N*-limit approximation to Eq. (32) as follows. We define a continuous eigenfunction $v:[0,1] \rightarrow \mathbb {C}$ such that $v_{i} = v(i/N)$. Then, taking the limit $N \rightarrow \infty $ to Eq. (32) with $\Delta _{ij} \approx \Delta _{j} \sim N(0, \delta ^{2})$, we obtain the continuous eigenvalue criterion for *λ* with respect to eigenfunction $v(x)$ given by

33 $$ \begin{aligned}[b] & \biggl[\lambda (\lambda +1) + \int _{-\infty }^{\infty }C_{\varOmega }( \Delta ) \bigl(\lambda + 1 - \tilde{\kappa }C_{0}(\Delta )H(\Delta )\bigr)\rho _{\delta }(\Delta ) \,d\Delta \biggr]v(x) \\ &\quad {} + \biggl( \int _{-\infty }^{\infty }C_{\varOmega }(\Delta )\bigl[ \tilde{\kappa } C_{0}( \Delta )H(\Delta ) - (\lambda +1)e^{-\lambda \tau ^{E}(\Delta )}\bigr] \rho _{\delta }(\Delta ) \,d\Delta \biggr) \int _{0}^{1} v(y) \,dy = 0 \end{aligned} $$

for every $x \in [0,1]$. Justification regarding the *N*-limit step to derive Eq. (33) is provided in Appendix B. Here, the only continuous eigenfunction solution to the above equations is the constant function $v(x) = 1$.^1^ Hence, Eq. (33) simplifies to the following *N*-limit eigenvalue equation for *λ*:

34 $$ \lambda = g \int _{0}^{\infty }C_{\varOmega }(\Delta ) \bigl(e^{-\lambda \tau ^{E}( \Delta )} - 1\bigr) \rho _{\delta }(\Delta ) \,d\Delta .$$

We claim that the synchronization state $(\varOmega , \delta ^{2})$ is (neutrally) stable given that for all $\lambda \in \mathbb {C}$ satisfying Eq. (34), $\operatorname {Re}(\lambda ) \leq 0$. Note that for all $\lambda = u + iv$ satisfying Eq. (34),

35 $$ \vert \lambda \vert \leq g \biggl( \vert I_{-1} \vert + \int _{0}^{\infty }e^{-u\tau ^{E}( \Delta )}\rho _{\delta }(\Delta ) \,d\Delta \biggr), $$

where $I_{-1} = \int _{0}^{\infty }C_{\varOmega }(\Delta )\rho (\Delta ) \, d \Delta $. By Eq. (35), if $u \rightarrow \infty $, $|\lambda | \leq g|I_{-1}|$, which is a contradiction. It follows that, as $|\lambda | \rightarrow \infty $, $\operatorname {Re}(\lambda ) \rightarrow -\infty $ for the distribution of eigenvalues *λ* satisfying Eq. (34). If $\kappa = 0$, then the frequency *Ω* solving Eq. (31) is (neutrally) stable if all eigenvalues $\lambda \in \mathbb {C}$ given by

36 $$ \lambda = g \cos \bigl(\varOmega \tau ^{0}\bigr) \bigl(e^{-\lambda \tau ^{0}} - 1\bigr) $$

have non-positive real parts $\operatorname {Re}(\lambda ) \leq 0$. As shown in [22], the non-plastic delay network synchronizes at *Ω* if and only if $\cos (\varOmega \tau ^{0}) > 0$.

At this point, the *N*-limit approximate eigenvalue Eq. (34) has done little to improve the *N*-dimensional criterion $\det M_{\lambda }= 0$, due to exponential blow-up that persists within the integrand term. We proceed to reduce the eigenvalue Eq. (34) into an exponential polynomial root equation as follows. Rescale $\lambda \rightarrow R \lambda $ by some radius $R > \kappa $,

37 $$ \lambda = R g \biggl( e^{-\lambda \tau ^{R}} \int _{0}^{\infty }C_{\varOmega }(\Delta ) e^{-\kappa \lambda \Delta /R} \rho _{\delta }(\Delta ) \,d\Delta - I_{-1} \biggr) $$

with rescaled small delay term $\tau ^{R} = \tau ^{0} / R$. Expressing the power series of the exponential *λ* term up to degree *M* in Eq. (37), we obtain the approximate exponential polynomial root equation

38 $$ P\bigl(\lambda \mid \varOmega , \delta ^{2}\bigr) + Q \bigl(\lambda \mid \varOmega , \delta ^{2}\bigr)e^{-\lambda \tau ^{R}} = 0 $$

with polynomials

39 $$ P\bigl(\lambda \mid \varOmega , \delta ^{2}\bigr) := \lambda + R g I_{-1},\qquad Q\bigl(\lambda \mid \varOmega , \delta ^{2}\bigr) := \sum_{m=0}^{M} I_{m} \lambda ^{m} ,$$

and power term coefficients

40 $$ I_{m} := \frac{(-1)^{m+1}R g}{m!} \biggl( \frac{\kappa }{R} \biggr)^{m} \int _{0}^{\infty }C_{\varOmega }(\Delta )\Delta ^{m} \rho _{\delta }(\Delta ) \,d\Delta ,\quad 0 \leq m \leq M .$$

Choosing a large radius $R > \kappa $, the rescaled delay term $\tau ^{R}$ and coefficients $I_{m}$ for larger degrees *m* become arbitrarily small. From this, we claim that the stability at $(\varOmega , \delta ^{2})$ is predominantly determined by the first few terms of the exponential power expansion. That is, the stability at synchronization state $(\varOmega , \delta ^{2})$ is determined by the finitely many polynomial roots $\lambda \in \mathbb {C}$ satisfying Eq. (38) with $\tau ^{R} \approx 0$ and some degree *M*. Denoting $\varLambda (\varOmega , \delta ^{2}) = \{\lambda _{1}, \ldots , \lambda _{M} \}$ as the roots of *M*-degree polynomial $P(\lambda \mid \varOmega , \delta ^{2}) + Q(\lambda \mid \varOmega , \delta ^{2})$, the synchronization state $(\varOmega , \delta ^{2})$ is (neutrally) stable given that

41 $$ E\bigl(\varOmega , \delta ^{2}\bigr) := \max \bigl\{ \operatorname {Re}(\lambda ): \lambda \in \varLambda \bigl(\varOmega , \delta ^{2} \bigr), \lambda \neq 0 \bigr\} < 0. $$

We note that these analytic results are reminiscent of what we obtained for two-dimensional systems in Sect. 4 with the cubic exponential polynomial equation $P_{\varOmega }(\lambda ) + Q_{\varOmega }(\lambda )e^{-\lambda \tau } = 0$ as defined by Eqs. (26) and (27).

As we experienced large scale fluctuations of stability term $E(\varOmega , \delta ^{2})$, we processed its value with either the sign function $\operatorname {sgn}(x)$ or the sign logorithm $\text{s.} \log (x)$ defined as

42 $$\begin{aligned}& \operatorname {sgn}(x) = \textstyle\begin{cases} 1, & x > 0, \\ -1, & x < 0, \\ 0, & x = 0, \end{cases}\displaystyle \end{aligned}$$

43 $$\begin{aligned}& \text{s.} \log (x) = \operatorname {sgn}(x)\log \bigl(1 + \vert x \vert \bigr). \end{aligned}$$

In Fig. 3(B), we plot $\operatorname {sgn}E(\varOmega , \delta ^{2})$ for all $\varOmega \in [\omega _{0} - g, \omega _{0} + g]$ and small $\delta > 0$. Stable regions are indicated where $\operatorname {sgn}E(\varOmega , \delta ^{2}) = -1$. In computing $E(\varOmega , \delta ^{2})$, eigenvalues satisfying $|\operatorname {Re}(\lambda )| > 10^{-8}$ were considered to be non-zero. We notice that a section of the level curve $\mathcal{R}(\varOmega , \delta ^{2}) = 0$ overlaps part of the stable region for $(\varOmega , \delta ^{2})$. Indeed, Fig. 3(C) plots the stability term $\text{s.} \log E(\varOmega , \delta ^{2})$ along all points $(\varOmega , \delta ^{2})$ on the implicit solution curve $\varOmega = \varOmega (\delta )$. As the plot shows, there is a small interval $(\delta _{l}, \delta _{r})$ such that $(\varOmega (\delta ), \delta )$ is stable for all $\delta \in (\delta _{l}, \delta _{r})$ such that $E(\varOmega , \delta ^{2}) < 0$. Figure 3(D) shows the transition of the eigenvalues $\lambda \in \mathbb {C}$ of $P(\lambda \mid \varOmega , \delta ^{2}) + Q(\lambda \mid \varOmega , \delta ^{2}) = 0$, corresponding to state $(\varOmega (\delta ), \delta ^{2})$ as *δ* leaves the interval $(\delta _{l}, \delta _{r})$. We see that the eigenvalues shift towards the right-side of the imaginary axis, which shows the transition from stability to instability along the solution curve $(\varOmega (\delta ), \delta ^{2})$.

The results above were obtained by setting the polynomial degree to $M = 3$ for $Q(\lambda \mid \varOmega , \delta ^{2})$, while the approximation failed for powers $M > 3$. In addition, it remains unresolved whether the distribution of eigenvalues $\lambda _{N}$ satisfying the *N*-dimensional Eq. (32) with some eigenvector $\vec{v} \in \mathbb {C}^{N}$ generally converge to the *N*-limit eigenvalues *λ* satisfying Eq. (33) with corresponding continuous eigenfunction $v:[0,1] \rightarrow \mathbb {C}$. That is, whether for each $\epsilon > 0$ there is some large *N* such that for all $\lambda _{N} \in \mathbb {C}$ satisfying Eq. (32) each at *N* dimensions, there exists some eigenvalue $\lambda \in \mathbb {C}$ satisfying Eq. (33) such that $|\lambda _{N} - \lambda | < \epsilon $. Integral equation theory has proven some relevant theorems. For instance, it can be proven [25] that there exists a non-zero eigenvalue $\lambda \in \mathbb {C}$ to Eq. (33) such that $\lambda _{N(k)} \rightarrow \lambda $ for some subsequence of eigenvalues $\lambda _{N(k)} \in \mathbb {C}$ satisfying Eq. (32) at dimension $N = N(k)$. Nevertheless, our *N*-limit analysis highlights an approximate region of points $(\varOmega , \delta ^{2})$ where a large-dimensional system of oscillators will synchronize. We demonstrate in the following section that this region aligns with synchronous behavior in numerical simulations.

## Comparison to numerical simulations

Here, we validate the theoretical analysis committed in the previous sections through comparisons with numerical simulations. We obtain the global frequency *Ω* and asymptotic phase-offsets $\phi _{i}$ numerically, and systematically compare them with their analytical counterparts. Given numerical solution $\theta _{i}(t)$, $i \leq N$ to the Kuramoto equation (1) with corresponding derivative solution $\theta _{i}'(t), i \leq N$, we can obtain the asymptotic frequencies for each oscillator by summing over the time interval $[t, t+h]$ and taking the limit $t \rightarrow \infty $. That is,

44 $$ \widehat {\varOmega _{i}} := \lim_{t \rightarrow \infty } \frac{1}{h} \int _{t}^{t+h} \theta _{i}'(t) \,dt $$

and estimate the global frequency *Ω* as the sample mean of individual frequencies

45 $$ \widehat {\varOmega} = \frac{1}{N} \sum _{i=1}^{N} \widehat {\varOmega _{i}} .$$

Likewise, we can numerically estimate the asymptotic phase-offsets $\phi _{i}$ for each oscillator by taking the difference $\widehat {\phi}_{i}(t) := \theta _{i}(t) - \widehat {\varOmega}t $ and defining the limit

46 $$ \widehat {\phi}_{i} := \lim_{t \rightarrow \infty } \frac{1}{h} \int _{t}^{t+h} \widehat {\phi}_{i}(t) \,dt .$$

After modding $\widehat {\phi}_{i}$ so that $\widehat {\phi}_{i} \in [-\pi , \pi )$, we can estimate the offset variance $\delta ^{2}$ by taking the sample variance

47 $$ \widehat {\delta ^{2}} := \frac{1}{N-1} \sum _{i=1}^{N} (\widehat {\phi}_{i} - \overline{\phi })^{2} $$

and the average (asymptotic) phase *ϕ̅* by taking the sample mean,

48 $$ \overline{\phi } := \frac{1}{N} \sum _{i=1}^{N} \widehat {\phi}_{i} .$$

If our solutions $\theta _{i}(t)$ synchronize towards some synchronous frequency *Ω*, offsets $\phi _{i}$, then *Ω̂*, $\widehat {\phi}_{i}$ are estimators for *Ω*, $\phi _{i}$, respectively. Numerically, we evaluate the estimators by taking the average over interval $[t, t+h]$ starting at some large time $t > 0$.

We set all baseline delays to be a unique value $\tau ^{0} > 0$, so that $\tau _{ij}(0) = \tau _{ij}^{0} = \tau ^{0}$. Since the plasticity rule Eq. (3) is an ordinary differential equation, it suffices for all delays to have an initial value at $t = 0$. Before $t = 0$, we set the phases $\theta _{i}(t)$ to be positioned in accordance to some initial frequency $\varOmega _{0}$ and initial phase-offsets $\phi _{i}^{0}$. That is, we define the linear initial function $\varphi _{i}(t) = \varOmega _{0} t + \phi _{i}^{0}$ and set $\theta _{i}(t) = \varphi _{i}(t)$ on $t \leq 0$.^2^ In order to have reasonably behaving solutions $\theta (t)$, we modify the function $\varphi _{i}(t)$ so that it satisfies the necessary condition

49 $$ \varphi _{i}'(0) = \frac{g}{N} \sum_{i=1}^{N} \sin \bigl(-\varOmega _{0} \tau ^{0} + \phi _{j}^{0} - \phi _{i}^{0}\bigr) $$

for all *i*. This adjustment is needed in order to avoid numerical discontinuities for $\theta _{i}'(t)$ at $t = 0$. For details, refer to Appendix C.

**Figure 4:**
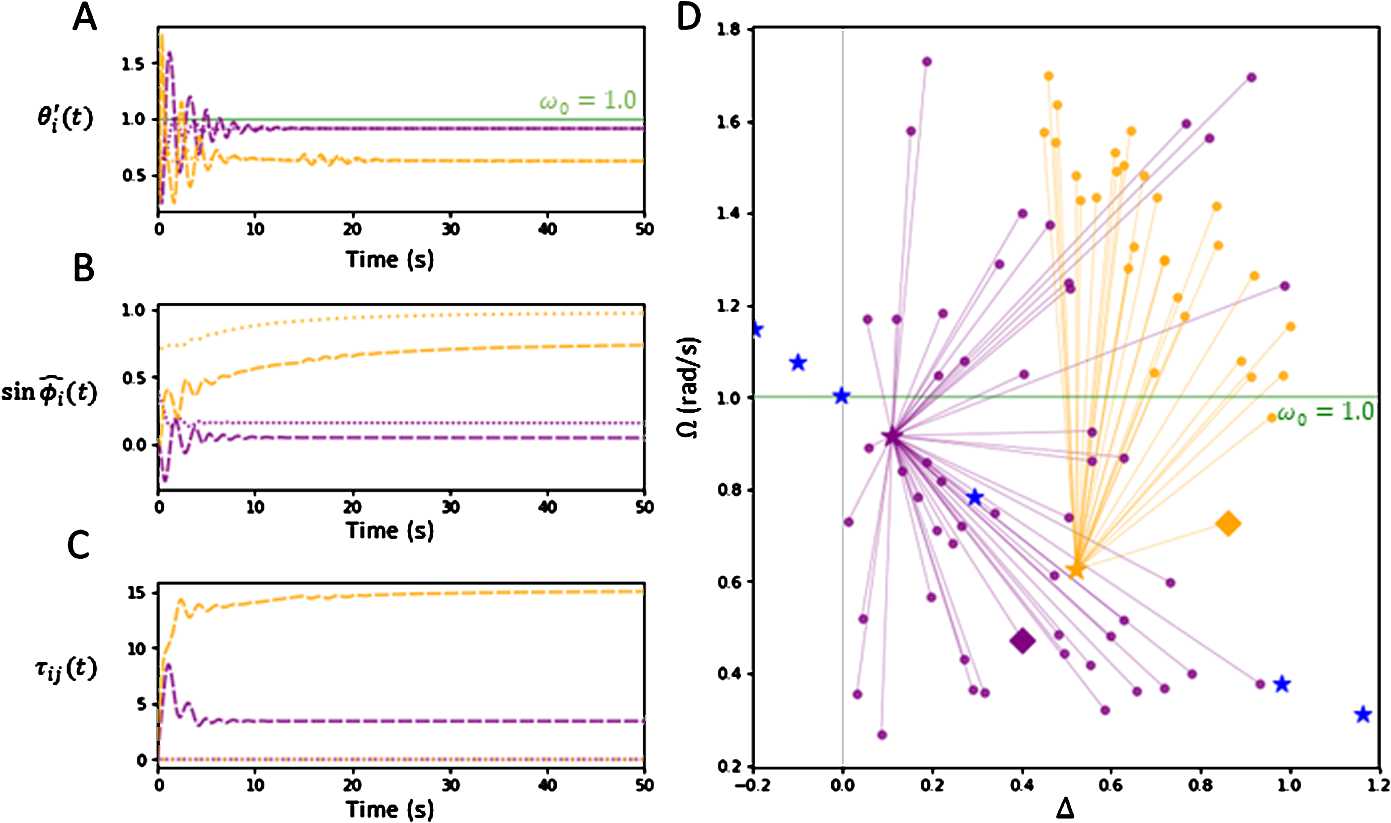
*Numerical plots for two-oscillator system*. For plots **A**, **B**, **C**, two trials with different initial conditions are graphed. Trial 1, 2 (purple, orange) starts with initial frequency and phase difference $(\varOmega _{0}, \Delta _{0}) = (0.473, 0.402), (0.727, 0.860)$, respectively. **A**. Plots of derivatives $\theta _{1}'(t)$, $\theta _{2}'(t)$ over time. For each trial, $\theta _{1}'(t)$ (dashed) and $\theta _{2}'(t)$ (dotted) converge to a common value *Ω̂* asymptotically. Each trial of oscillators entrain to a different frequency, implying the existence of multiple synchronization frequencies. **B**. Plots of sine phases $\sin (\widehat {\phi}_{i}(t))$, where $\widehat {\phi}_{i}(t) = \theta _{i}(t) - \widehat {\varOmega}t$. For both trials, the oscillators asymptotically phase-lock with $\widehat {\phi}_{i}(t) \rightarrow \widehat {\phi}_{i}$, $i=1$ (dotted), $i=2$ (dashed). The phase-lock difference $\widehat {\Delta}_{12} = \widehat {\phi}_{2} - \widehat {\phi}_{1}$ is also different for the two trials. **C**. Plots of adaptive delays $\tau _{12}(t)$ (dashed), $\tau _{21}(t)$ (dotted) over time. For each trial, delay $\tau _{12}(t)$ converges to some positive equilibrium $\tau ^{E}$, and delay $\tau _{21}(t)$ decays to 0. **D**. Plots showing where two-oscillators with randomized initial conditions $(\varOmega _{0}, \Delta _{0})$ (orange) synchronize towards in terms of asymptotic frequency and phase-offset $(\widehat {\varOmega}, \widehat {\Delta}_{12})$ (magenta) across 80 trials. Each of the two trials in **A**, **B**, **C** have their initial condition plotted as a diamond marker of matching colour. Theoretical synchronization states $(\varOmega , \Delta _{12})$ given by Eqs. (20) and (22) are also plotted (blue). Trials converge to two states $(\widehat {\varOmega}, \widehat {\Delta}_{12}) = (0.625, 0.522), (0.916, 0.111)$, which align with the two theoretically stable states shown in Fig. 2. The parameters used for all plots are $\alpha _{\tau }= 0.5$, $\varepsilon = 0.01$, $g = 1.5/2$, $\kappa = 30$, $\omega _{0} = 1.0$, $\tau ^{0} = 0.1~\text{s}$

Figure 4 plots the results of a series of numerical simulations in the reduced two-oscillator network as set up in Sect. 4. All trials were run from 0–200 s, with estimated values *Ω̂*, $\widehat {\phi}_{i}$ obtained by averaging the arrays over the last 20 seconds. The same parameter values used in Fig. 2 were applied here when running the numerical trials. We demonstrate the existence of two stable synchronization states with different frequencies. Figure 4(A), (B), (C) shows the asymptotic behaviour of two trials (purple, orange) that started with different initial functions, plotting over the first 50 seconds. Figure 4(A) plots the derivative arrays $\theta _{1}'(t)$, $\theta _{2}'(t)$ of each trial. We observe in Fig. 4(A) that each pair of oscillator frequencies entrain to a common frequency over time. The two trials converge to different common frequencies, estimated to be $\widehat {\varOmega} = 0.916$ (purple lines) and $\widehat {\varOmega} = 0.625$ (orange lines), respectively. Figure 4(B) plots the offset arrays $\sin \widehat {\phi}_{1}(t)$, $\sin \widehat {\phi}_{2}(t)$ of each trial. For each trial, the two-oscillators become phase-locked as $\widehat {\phi}_{2}(t) - \widehat {\phi}_{1}(t)$ converges asymptotically to estimated constant differences $\widehat {\Delta}_{12} = 0.111$ (purple lines) and $\widehat {\Delta}_{12} = 0.523$ (orange lines), where $\widehat {\Delta}_{12} = \widehat {\phi}_{2} - \widehat {\phi}_{1}$. Figure 4(C) plots the connection delays $\tau _{12}(t)$, $\tau _{21}(t)$ over time. By choosing our plasticity gain $\kappa = 30 \gg 0.1 = \tau ^{0}$, it follows that for both trials, $\tau _{12}(t)$ converges to some positive equilibrium delay $\tau ^{E}$ and $\tau _{21}(t)$ decays to 0.

The above results imply that there exist at least two stable synchronization states, and that the frequency the system converges towards is dependent on the initial functions $\varphi _{1}(t)$, $\varphi _{2}(t)$. To provide further confirmation towards this proposition, the trials shown in Fig. 4 were repeated 80 times randomized across sampled initial frequency $\varOmega _{0} \in [\omega _{0} - g, \omega _{0} + g]$ and initial phase difference $\Delta _{0} \in (0,1)$. The point $(\varOmega _{0}, \Delta _{0})$ (circle markers) defines the initial functions $\varphi _{1}(t) = \varOmega _{0} t$, $\varphi _{2}(t) = \varOmega _{0} t + \Delta _{0}$. The theoretically stable states corresponding to frequencies $\varOmega = \varOmega _{1}$ and $\varOmega = \varOmega _{3}$ are plotted as orange and purple stars, respectively, which are both close to the asymptotic frequency of the matching coloured trial discussed above. The rest of the theoretical synchronous states given by the roots of Eq. (23) are plotted as blue stars. Each trial’s solution arrays synchronized near one of the two stable states, as shown by the connecting coloured lines. The matching colours indicate which of the two stable frequencies $\varOmega _{i}$ the trial’s solution arrays synchronized towards, such that the $| \widehat {\varOmega} - \varOmega _{i} | < 5 \times 10^{-3}$. Each of the two trials graphed in Fig. 4(A), (B), (C) are also plotted in Fig. 4(D) with a diamond marker of matching colour. Convergence of the points is suggestive of a separatrix curve between the basins of attraction of both stable fixed points. It was also observed that the system synchronized towards the frequency $\varOmega = \varOmega _{3}$ faster than $\varOmega = \varOmega _{1}$, which suggests that the state $\varOmega = \varOmega _{3}$ has a greater force of attraction. Hence, the experimental results align with the analysis outlined in Sect. 4. We draw the conclusion that in our reduced two-oscillator system, plastic delays are able to generate multiple synchronization states in comparison to non-plastic delays.

**Figure 5:**
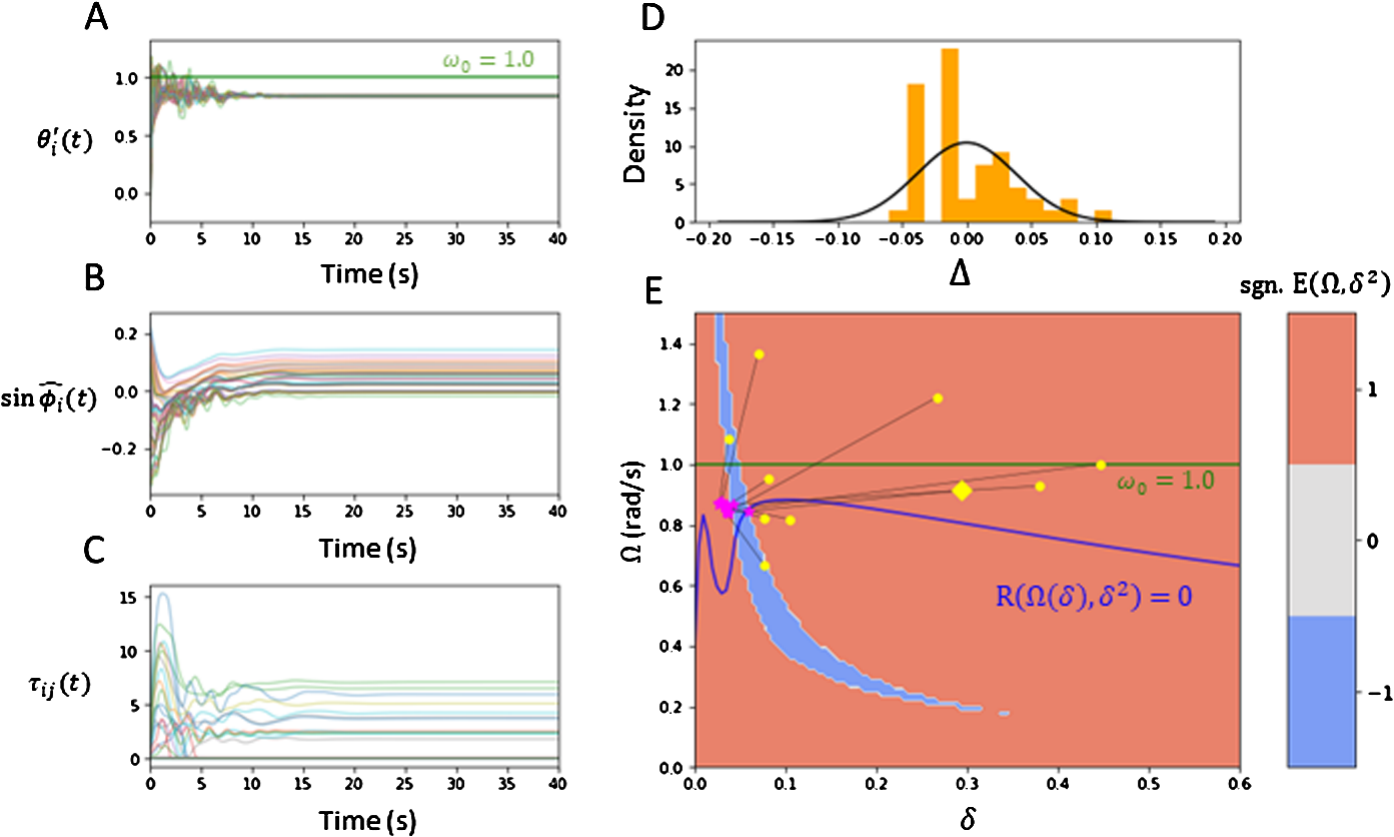
*Numerical plots of* *N-oscillator system*. **A**. Plots of derivatives $\theta _{i}'(t)$ over time. We see that all $\theta _{i}'(t)$ converge to a common frequency, estimated to be $\widehat {\varOmega} = 0.839$. **B**. Plots of sine phases $\sin (\widehat {\phi}_{i}(t))$, where $\widehat {\phi}_{i}(t) = \theta _{i}(t) - \widehat {\varOmega}t$. All oscillators appear to asymptotically phase-lock to one another. **C**. Plots of a sample of 50 adaptive delays $\tau _{ij}(t)$ over time. Some delays $\tau _{ij}(t)$ converge to some positive equilibrium $\tau _{ij}^{E}$, while others decay to 0. **D**. Density of centralized phase-offsets $\widehat {\Delta}_{i} = \widehat {\phi}_{i} - \overline{\phi }$, which was assumed to be Gaussian. The Gaussian curve $N(0, \widehat {\delta}^{2})$ (black line) is fit over the density. **E**. Plot showing oscillators with randomized initial frequency and phase deviation $(\varOmega _{0}, \delta _{0})$ (yellow) synchronizing towards respective estimated asymptotic frequency and phase deviation $(\widehat {\varOmega}, \widehat {\delta})$ (magenta star) across 10 trials. The yellow diamond refers to the trial plotted in **A**, **B**, **C**. The numerical values are plotted directly over Fig. 3(B). As shown, the network synchronizes near the theoretical stable region. The parameters used were $N = 50$, $\varepsilon =0.01$, $\alpha _{\tau }= 0.1$, $g = 1.5$, $\kappa =80$, $\omega _{0} = 1.0$, $\tau ^{0} = 0.1~\text{s}$

Figure 5 provides the numerical results of a similar experiment performed as in Fig. 4 with a large-dimensional network $N = 50$ and all-to-all network. All trials were run from 0-100 s, with estimated values *Ω̂*, $\widehat {\phi}_{i}$ obtained by averaging the arrays over the last 10 seconds. The same parameter values are used as in Fig. 3. The initial function $\varphi (t)$ for each trial was set up by choosing some frequency $\varOmega _{0} \in [\omega _{0} - \frac{g}{4}, \omega _{0} + \frac{g}{4}]$ and deviation $\delta _{0} \in (0, 0.5)$. The initial phases $\phi _{i}^{0}$ were i.i.d. sampled uniformly from the interval $[-\sqrt{3}\delta _{0}, \sqrt{3}\delta _{0}]$. The *i*th oscillator was equipped with the initial linear function $\varphi _{i}(t) = \varOmega _{0}t + \phi _{i}^{0}$. Figure 5(A), (B), (C), (D) graphs a single numerical trial with $\varOmega _{0} = 0.913$, $\delta _{0} = 0.295$. Figure 5(A) plots the derivative arrays $\theta _{i}'(t)$, which we note converges to some constant frequency estimated as $\widehat {\varOmega} = 0.839$. Figure 5(B) plots the offset arrays $\sin \widehat {\phi}_{i}(t)$, which shows that each $\widehat {\phi}_{i}(t)$ converges to some constant phase-offset estimated by $\widehat {\phi}_{i}$. Hence, the oscillators become asymptotically phase-locked under distributed offsets with estimated variance $\widehat {\delta}^{2} = 0.050^{2}$. Figure 5(C) plots a sample of 50 adaptive delays $\tau _{ij}(t)$, which become part of the positive equilibrium distribution $\tau _{ij}^{E} > 0$ or decay to 0. Figure 5(D) plots the density of centered phases $\widehat {\Delta}_{i} = \widehat {\phi}_{i} - \overline{\phi }$. As we assumed that the centralized phase-offsets follow a Gaussian distribution, we perform a normality test on the numerical asymptotic offsets $\widehat {\Delta}_{i}$. The Shapiro–Wilk test for non-normality returned a p-value of 0.005 for $\widehat {\Delta}_{i}$, which suggests another distribution would be more accurate. Visually, a Gaussian curve $N(0, \widehat {\delta}^{2})$ (black line) is fit over the density in Fig. 5(D). Nevertheless, the relevance of the Gaussian approximation, which greatly simplifies the analysis, becomes apparent as the numerical and analytical results nearly coincide. Other approximations could be used to facilitate the analysis further, and are left for future work.

The numerical simulation, as presented above, was repeated 10 times with randomized initial conditions $(\varOmega _{0}, \delta _{0}^{2})$. For each trial, $\varOmega _{0}$, $\delta _{0}$ was sampled uniformly from intervals $[\omega _{0} - \frac{g}{4}, \omega _{0} + \frac{g}{4}]$ and $(0, 1)$, respectively. Figure 5(E) plots the following convergence results. Each trial with initial condition $(\varOmega _{0}, \delta _{0}^{2})$ (yellow markers) synchronized near the respective estimated point $(\widehat {\varOmega}, \widehat {\delta}^{2})$ (magenta markers). We can see that every trial synchronized at approximately the same state $(\varOmega , \delta ^{2})$. To determine whether the numerical results align with the analysis discussed in Sect. 5, the numerical values were plotted on top of Fig. 3(B). We observe that for each trial, the network synchronizes near the portion of the level curve $\mathcal{R}(\varOmega , \delta ^{2}) = 0$ within the stable region where $E(\varOmega , \delta ^{2}) < 0$. Hence, the numerical experiment for $N = 50$ generated results that validates the theoretical *N*-limit stability analysis in Sect. 5.

## Neuroscience application: resilience to injury with sparse and uniform connectivity

One of the most salient examples of white matter plasticity comes from neuroimaging in the presence of a lesion. In these cases, white matter remodeling takes place in order to restore and maintain function, a process that notably impacts neural synchronization [26]. Let us now investigate whether the plasticity mechanism can be used to stabilize phase-locked states in the presence of network damage. That is, we would like to know whether changes in time delays can be used to compensate for a reduction in effective connectivity and make the global synchronous state more resilient. To investigate this problem, we model injury as a loss in connections $a_{ij}$. Defining $\gamma \in [0,1]$ as the insult index, we introduce here the sparse synaptic connectivity weights given by

50 $$ a_{ij} = \textstyle\begin{cases} 1, & p_{ij} \geq \gamma , \\ 0, & p_{ij} < \gamma , \end{cases} $$

where $p_{ij}$ is a uniformly distributed i.i.d. sampling on $[0,1]$. *γ* represents the connectivity damage through an increase in the sparseness of network connections. Note that if $\gamma = 0$, then we obtain the all-to-all connection topology $a_{ij} \equiv 1$ corresponding to no injury in the system. If $\gamma = 1$, we have the trivial system $a_{ij} = 0$ which means no signals between the oscillators occur. For any *γ* we have the probability to retain the connection $\mathbb {P}(a_{ij} = 1) = 1 - \gamma $. Without plasticity, the mean phase dynamics for $N \rightarrow \infty $ is given by

51 $$ \varOmega = \omega _{0} + (1 - \gamma )g \sin \bigl(- \varOmega \tau ^{0}\bigr); $$

see Eq. (31). By observation, one can see that we are in the presence of the same network dynamics, but with an effective coupling coefficient given by $g_{\text{eff}} := (1 - \gamma )g$. Thus, damage simply reduces the net coupling. To demonstrate this point, we start with a strong coupling parameter and decrease it until stability is either lost or preserved by plasticity. According to Eq. (51), $\varOmega \rightarrow \omega _{0}$ as $\gamma \rightarrow 1$. Hence, if the baseline lag $\tau ^{0}$ is chosen such that $\cos (\varOmega \tau ^{0}) \geq 0$ and $\cos (\omega _{0}\tau ^{0}) < 0$, the network without plastic delays is susceptible to injury destabilizing the synchronous state.

**Figure 6:**
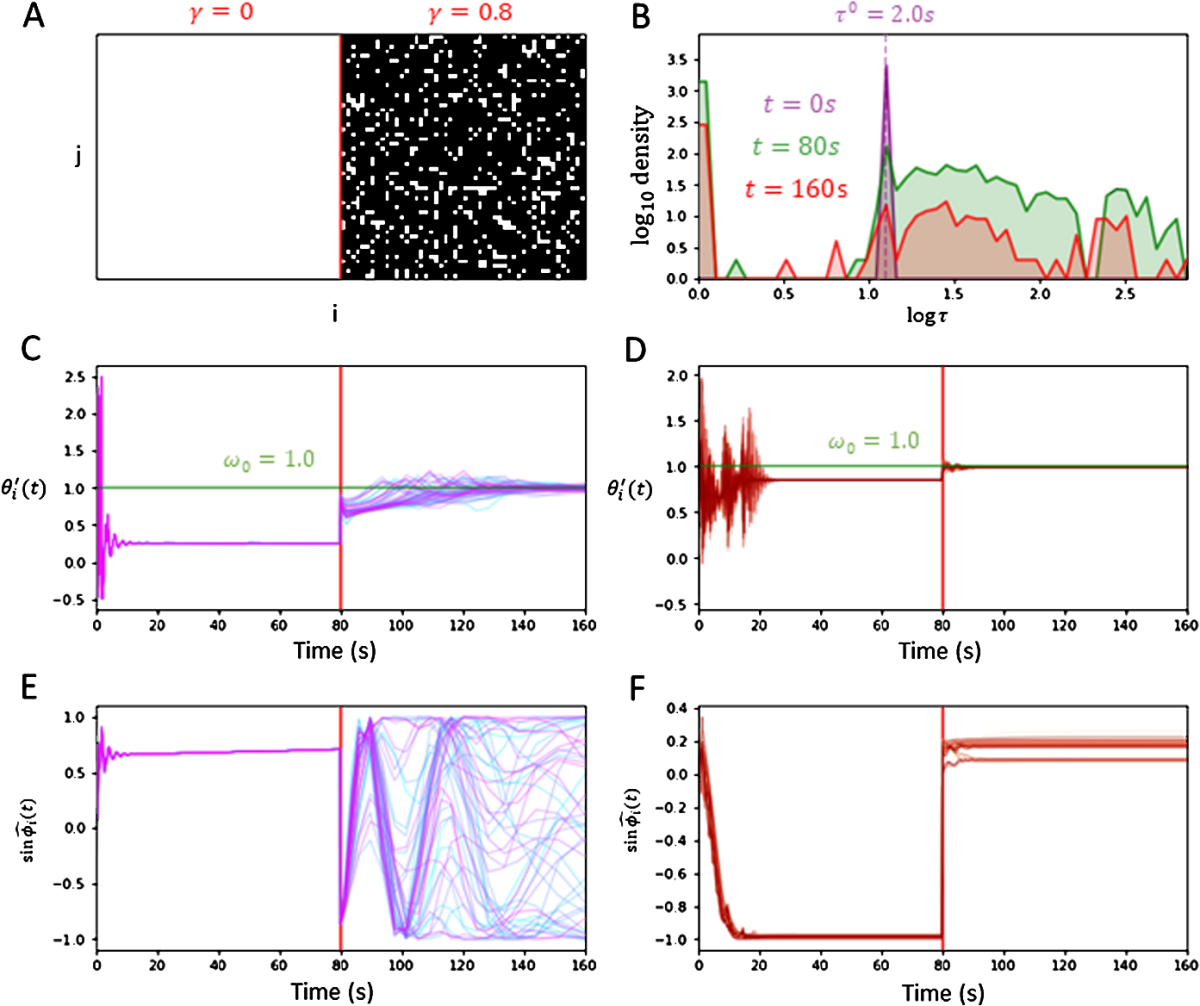
*Comparing resilience against injury between plastic and non-plastic delays*. Injury towards the connection topology $a_{ij}$ is introduced at $t_{\mathrm{inj}} = 160~\text{s}$ (red line). **A**. Plots of the connection matrix $A = (a_{ij})$ before injury ($\gamma = 0$) and after injury ($\gamma = 0.8$), with $a_{ij} = 1, 0$ indicated in white, black, respectively. **B**. The log histogram plots of delays at initial time $t = 0~\text{s}$ (purple), midtime before injury $t = 160~\text{s}$ (green), and at the end time following injury $t = 320~\text{s}$ (red). The delays become distributed away from $\tau _{ij} = \tau ^{0}$ to either some largely varying equilibrium delays $\tau _{ij}^{E} > 0$ or decay to 0. Following injury, there are fewer delays available to stabilize the synchronous network. **C**, **D**. Plots of derivatives $\theta _{i}'(t)$ over time, without and with plasticity, respectively. Both networks entrain in frequency pre-injury. Following injury, both networks entrain to a new frequency closer to $\omega _{0}$. **E**, **F**. Plots of $\sin \widehat {\phi}_{i}(t)$ over time, without and with plasticity, respectively. Following injury, the oscillators with plastic delays are able to coherently phase-lock within close proximity to each other, while the network without plastic delays remain in a non-convergent state. The parameters used were $N = 50$, $\varepsilon =0.01$, $\alpha _{\tau }= 1.0$, $g = 1.5$, $\kappa = 80$, $\omega _{0} = 1.0$, and $\tau ^{0} = 2.0~\text{s}$

We ran numerical simulations by applying similar parameter values as Sect. 6 while introducing injury $\gamma = 0.8$ to the connections at time $t = t_{\mathrm{inj}}$, set at $t_{\mathrm{inj}} = 80~\mbox{s}$. The initial condition of the network was fixed at $(\varOmega _{0}, \delta _{0}) = (\omega _{0}, 0.25)$ for all trials. Figure 6(A) shows the destruction of existing connections following injury, comparing connection grids for $a_{ij}$ before and after $t_{\mathrm{inj}}$. Figure 6(B) shows the distribution of the delays $\tau _{ij}(t)$ with existing connections $a_{ij} = 1$ at timestamps $t = 0~\mbox{s}$, $t = 79~\mbox{s}$ (pre-injury), $t = 160~\mbox{s}$ (post-injury). With fewer connections available, the ability for the surviving delays to adjust themselves are crucial in re-stabilizing the system’s synchrony. Figure 6(C) (no gain) and Fig. 6(D) (with gain) show that both networks entrain to a global frequency successfully before and after inflicted injury towards the network. The entrainment frequencies pre-injury $\varOmega _{\text{pre}} \approx \widehat {\varOmega}_{\text{pre}}$ and post-injury $\varOmega _{\text{post}} \approx \widehat {\varOmega}_{\text{post}}$ were estimated by taking the average of $\theta _{i}'(t)$ at times 148–160 s and 304–320 s, respectively, for each $i \leq N$. Figure 6(E) (no gain) and Fig. 6(F) (with gain) plots the sine phase-offsets $\sin \widehat {\phi}_{i}(t)$ over time, given by

52 $$ \widehat {\phi}_{i}(t) = \textstyle\begin{cases} \theta _{i}(t) - \widehat {\varOmega}_{\text{pre}} t, & t < t_{\mathrm{inj}}, \\ \theta _{i}(t) - \widehat {\varOmega}_{\text{post}} t, & t \geq t_{\mathrm{inj}}. \end{cases} $$

Following injury, from Fig. 6(E) the network without adaptive delays collectively falls out of phase. In contrast, Fig. 6(F) shows the network with adaptive delays demonstrating resilience against the injury as most oscillators are able to collectively phase-lock within close proximity to each other.

**Figure 7:**
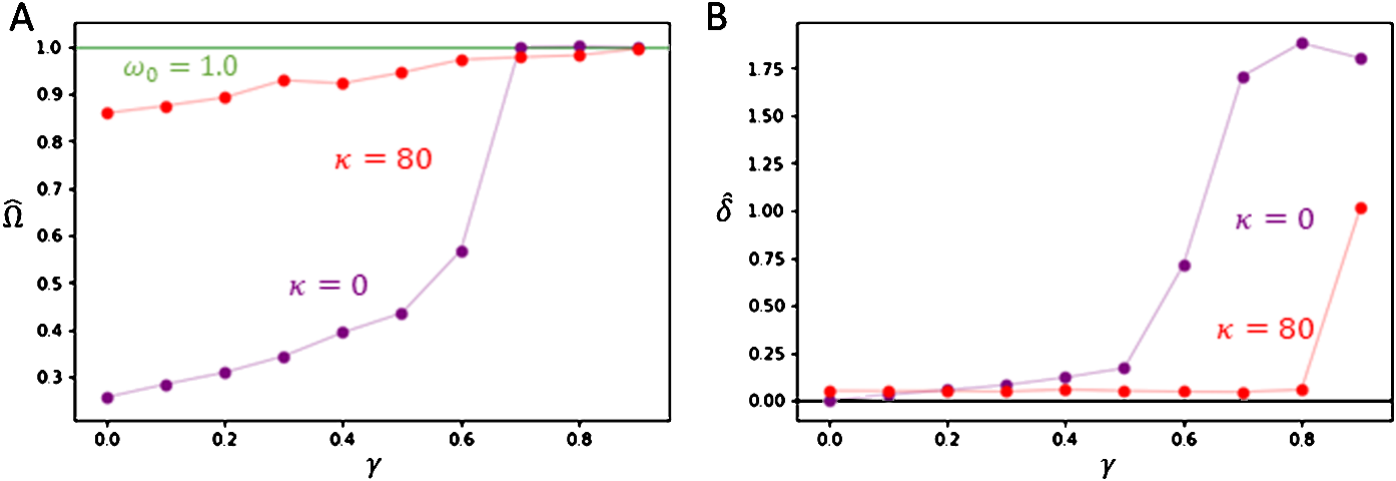
*Comparing of post-injury network asymptotic behaviour with increasing injury between plastic and non-plastic delays*. Each numerical trial was run over 320 s, and all asymptotic values were evaluated by averaging over the final 16 s. Injury was introduced at $t = 160~\text{s}$. Both plots show trials with adaptive delays (red) and fixed delays (purple). **A**. Plot of post-injury asymptotic frequency *Ω̂* for trials with injury $\gamma > 0$. As *γ* increases, $\widehat {\varOmega} \rightarrow \omega _{0}$. **B**. Plot of post-injury asymptotic standard deviation *δ̂* for trials with injury $\gamma > 0$. As *γ* increases, *δ̂* has significant increase for trials without plasticity, while *δ̂* remains relatively small for trials with plasticity until $\gamma = 0.9$. The parameters used were $N = 50$, $\varepsilon =0.01$, $\alpha _{\tau }= 1.0$, $g = 1.5$, $\kappa = 80$, $\omega _{0} = 1.0$, and $\tau ^{0} = 2.0~\text{s}$

Figure 7 examines the effect of gradually increasing the severity of injury towards the system’s global frequency $\varOmega \approx \widehat {\varOmega}$ and its phase-offset variance $\delta ^{2} \approx \widehat {\delta}^{2}$. For each trial at injury *γ*, the same initial condition and parameters were used as in Fig. 6. Figure 7(A) shows that connection loss generally leads to the system’s synchronization frequency *Ω* becoming closer to the natural frequency $\omega _{0}$. Figure 7(B) plots the estimated phase standard deviation *δ̂* with respect to increasing injury. The network without plastic delays exhibits a significant loss in coherent synchrony with increasing $\widehat {\delta} > 0$ as more connections are lost. In contrast, the network equipped with adaptive delays persistently displays phase coherence with $\widehat {\delta} \ll 1$ until higher injury levels *γ*.

## Discussion

Our goal was to provide a mathematical framework that captures the synchronizing properties of networks with adaptive delays. We sought to implement the activity-dependent property of myelinated connection delays by modifying the Kuramoto model as proposed in [9]. The focus was to determine whether such adaptive delays significantly improve the oscillatory system’s ability to become in-phase and to entrain to a global frequency. Given that this is the case, the results of the model’s study reinforces the proposition that myelin plasticity is essential in maintaining the synchrony in the developing or injured brain.

White matter plays a critical role in maintaining brain function through the coordination of neural dynamics across multiple temporal and spatial scales. Recent evidence has shown that through the action of glia, white matter properties evolve continuously in time. Specifically, conduction velocity within and across brain areas is adjusted to promote efficient neural signaling. While the mechanism remains poorly understood, the consequences of such plastic processes on brain dynamics and synchronization can be readily examined and characterized using simplified mathematical models.

To accomplish this, we here examined the influence of adaptive conduction delays on the synchronization of neural oscillators. We developed a repertoire of mathematical tools to better examine the stability of phase-locked solutions. In theory, we derived implicit equations for the global frequency *Ω* and eigenvalues $\lambda \in \mathbb {C}$ that provide a rigorous criterion for the stability around the synchronous state in two-dimensional and large-dimensional settings. Based on our model, flexibility in the white matter structure introduces an additional corrective dynamic next to the phase interactions that can further drive the network’s phase alignment. Higher stability with adaptive delays was demonstrated as the Kuramoto model had higher resilience against injury perturbations. However, adaptive delays improve the system’s synchronous features only when the delays adjust with a sufficiently high degree of plasticity, as represented by the plasticity gain *κ*.

There are many limitations in the prototypical model we used and its corresponding results. Myelination is bound by many physiological constraints, some of which remain uncertain [4]. It is established that white matter restructures itself in response to ongoing neural activity [8]. We primarily incorporated this fact in our plasticity rule in a manner that promotes local synchrony. Indeed, each connection delay changes at a rate proportional to the sine of the oscillator’s phase difference. This rule remains a tentative construction, as more research is needed to develop more biological relevant models in activity-dependent myelination. In addition, the use of phase oscillators to model local neural dynamics remain limited and is relevant mostly in the context of large-scale neural systems. In our analysis, we relied heavily on i.i.d. parametrical frameworks in order to establish our *N*-limit approach, which may not be feasible as network elements are correlative in nature.

Moving forward, we hope to build upon our analysis alongside newly found experimental results pertaining to myelin. Despite its shortcomings, the mathematical approaches used and its results can potentially be applied to more complex, biological relevant models. The conduction delays $\tau _{ij}(t) \propto c_{ij}^{-1}(t)$ can be alternatively modelled with respect to a system of adaptive conduction velocities $c_{ij}(t)$. In the realm of temporal equations, other parametric avenues have yet to be explored. For instance, the delays $\tau _{ij}(t)$ can exhibit slow convergence by setting the rate constant $\alpha _{\tau }\ll 1$. The aforementioned concepts are some proposed examples that may further lead to uncharted dynamics in the scope of neurocomputational models.

## Appendix A: Existence, uniqueness, and linearization

We sketch out the arguments needed to justify the existence, uniqueness, and stability analysis of our proposed Kuramoto model with adaptive delays. Our model can be expressed as an $N + N^{2}$ system of oscillators $\theta (t) = (\theta _{i}(t))_{1 \leq i \leq N} \in \mathbb {R}^{N}$ and delays $\tau (t) = (\tau _{ij}(t))_{1 \leq i,j \leq N} \in \mathbb {R}_{+}^{N \times N}$, where $\mathbb {R}_{+} := [0, \infty )$, defined by the state-dependent functional differential equations

53 $$\begin{aligned}& \dot{\theta }(t) = F\bigl(\theta _{t}, \tau (t)\bigr), \end{aligned}$$

54 $$\begin{aligned}& \dot{\tau }(t) = G\bigl(\theta (t), \tau (t)\bigr), \end{aligned}$$

where $\theta _{t} \in C := ([-r,0], \mathbb {R}^{N})$ defined by $\theta _{t}(s) = \theta (t+s)$, $s \in [-r,0]$, and *C* is the space of all continuous functions from interval $[-r,0]$ for some $r > 0$. Here, $F: C \times \mathbb {R}_{+}^{N \times N} \rightarrow \mathbb {R}^{N}$ is the function whose *i*th co-ordinate $F_{i}(\theta _{t}, \tau (t))$ is given by Eq. (1) and $G: \mathbb {R}^{N} \times \mathbb {R}_{+}^{N \times N} \rightarrow \mathbb {R}_{+}^{N \times N}$ is the function whose *ij*th co-ordinate $G_{ij}(\theta _{i}(t), \theta _{j}(t), \tau _{ij}(t))$ is given by Eq. (3). We note that, from Eq. (3), each delay solution $\tau (t)$ is bounded for all $t \geq 0$. This is clear since given $\tau _{ij}(t) > \tau ^{0} + \kappa $, we must have $\tau _{ij}'(t) < 0$. Hence, it suffices to define the space *C* with any $r > \tau ^{0} + \kappa $.

Denote $C^{1} \subset C$ to be the subspace of all continuously differentiable functions. Let $\theta _{0}(s) = \varphi (s)$ for some $C^{1}$ initial function $\varphi : [-r,0] \rightarrow \mathbb {R}^{N}$ and $\tau (0) = \tau ^{0} \in \mathbb {R}_{+}^{N \times N}$ be the initial delays. Since Eq. (54) is a system of ordinary differential equations, $\tau (t)$ is independent of initial values on $t < 0$. Hence, we represent an initial function for $\tau (t)$ with an initial point $\tau ^{0} \in \mathbb {R}_{+}^{N \times N}$. Existence and uniqueness of solution $(\theta (t), \tau (t))$ follows from theorems in [18] given that both *F*, *G* are continuously differentiable functions over $(\varphi , \tau ^{0}) \in C^{1} \times \mathbb {R}_{+}^{N \times N}$. The *i*th co-ordinate $F_{i}(\varphi , \tau ^{0})$ is a linear combination of terms $\sin (\varphi _{j}(\tau _{ij}^{0}) - \varphi _{i})$, which is continuously differentiable over $\varphi \in C^{1}$. *G* is continuously differentiable given the Heaviside function $H(\tau )$ is smooth. The Heaviside function used throughout the paper is constructed as follows. Setting some arbitrarily small $\varepsilon > 0$, we define the smooth mollifier function $h: \mathbb {R}\rightarrow \mathbb {R}$ by

55 $$ h(x) = \textstyle\begin{cases} e^{-(x-1)^{-2}} \cdot e^{-(x+1)^{-2}} & \text{if } -1 < x < 1, \\ 0 & \text{otherwise}. \end{cases} $$

Then our smooth Heaviside function $H(\tau )$ is defined as

56 $$ H(\tau ) = \textstyle\begin{cases} \frac{\int _{0}^{\tau }h(-1 + 2\varepsilon ^{-1}s) \,ds}{\int _{0}^{\varepsilon }h(-1 + 2\varepsilon ^{-1}s) \,ds} & \text{if } \tau > 0, \\ 0 & \text{otherwise}. \end{cases} $$

Then each co-ordinate $G_{ij}$ is smooth with respect to terms $\varphi _{i}$, $\varphi _{j}$, $\tau ^{0}$.

Linearization and stability of our system around the synchronized state $(\varOmega , \boldsymbol {\phi })$ can be justified as follows. We can express Eqs. (53) and (54) under perturbation terms $(\epsilon (t), \eta (t))$ of $(\theta (t), \tau (t))$ defined by Eqs. (7) and (8). That is, consider the autonomous equations

57 $$\begin{aligned}& \dot{\epsilon }(t) = \tilde{F}\bigl(\epsilon _{t}, \eta (t)\bigr), \end{aligned}$$

58 $$\begin{aligned}& \dot{\eta }(t) = \tilde{G}\bigl(\epsilon (t), \eta (t)\bigr), \end{aligned}$$

where the *i*th co-ordinate of $\tilde{F}(\epsilon _{t}, \eta (t))$ is given by Eq. (10) and $\tilde{G}(\epsilon (t), \eta (t)) = G(\varOmega t + \boldsymbol {\phi }+ \epsilon (t), \tau ^{E} + \eta (t))$. Then, if $(\varOmega , \boldsymbol {\phi })$ satisfies Eq. (5), $\tilde{F}(0,0) = \tilde{G}(0,0) = 0$. Hence, the stability of $(\theta , \tau )$ around $(\varOmega , \boldsymbol {\phi })$ depends on the linearization of *F̃*, *G̃* around $(0,0)$. One can compute that the corresponding linearized system $(\dot{\epsilon }(t), \dot{\eta }(t)) = L(\epsilon (t), \eta (t))$ is given by Eqs. (9) and (11). To prove that this linearization is valid and provides the eigenvalue stability criteria $\det M_{\lambda }= 0$, we can replicate the proof for Theorem 2.1 in [20]. Indeed, *F̃*, *G̃* are composed of Lipschitz continuous terms that allow the steps to be valid for our perturbation Eqs. (57) and (58).

## Appendix B: Derivation of *N*-limit equations

Here, we justify the limit steps taken in Sect. 5 to derive the limit-*N* equations for global frequency *Ω* and stability eigenvalues *λ* around the synchronous state. First, denote $\mathbb {E}g(X)$ as the expectation of random variable $g(X)$. That is, given *X* has distribution $\rho (x)$,

59 $$ \mathbb {E}g(X) = \int g(x) \rho (x) \,dx. $$

The main proposition we applied is as follows.

### Theorem 1

*Let* $(v_{k})_{k \geq 1}$*be a bounded sequence of real numbers*, *and* *X* *be a random variable such that* $\mathbb {E}X = 0$, $\mathbb {E}|X| < \infty $. *Suppose* $(X_{k})_{k \geq 1}$*is an i*.*i*.*d*. *sequence of random variables such that each* $X_{i}$*has the same distribution as* *X*. *Then*

60 $$ \frac{1}{N} \sum_{k=1}^{N} v_{k} X_{k} \rightarrow 0 $$

*almost surely as* $N \rightarrow \infty $.

### Proof

For some $M > 0$, $|v_{k}| < M$ for all *k*. By the classic Strong Law of Large Numbers,

61 $$ \Biggl\vert \frac{1}{N} \sum _{k=1}^{N} v_{k}X_{k} \Biggr\vert \leq M \Biggl\vert \frac{1}{N} \sum _{k=1}^{N} X_{k} \Biggr\vert \rightarrow 0 $$

almost surely as $N \rightarrow \infty $. □

What directly follows from Theorem 1 is the following limit result, which we utilize to derive the limit-*N* equations for the global frequency *Ω* and the stability eigenvalues *λ*.

### Corollary 1

*Let* $(v_{k})_{k \geq 1}$*be a bounded sequence in* $\mathbb {C}$, *and* *X* *be a random variable such that* $\mathbb {E}|X| < \infty $. *Suppose* $(X_{k})_{k \geq 1}$*is an i*.*i*.*d*. *sequence of random variables such that each* $X_{i}$*has the same distribution as* *X*. *Then*

62 $$ \frac{1}{N} \sum_{k=1}^{N} v_{k} X_{k} \rightarrow \overline{v} \mathbb {E}X, $$

*where* *v̅* *is the well*-*defined asymptotic sample mean of the coefficients*:

63 $$ \overline{v} = \lim_{N \rightarrow \infty } \frac{1}{N} \sum_{k=1}^{N} v_{k}. $$

*In particular*, *if* $v:[0,1] \rightarrow \mathbb {C}$*is a continuous function and* $v_{k} = v(k/N)$, *then*

64 $$ \frac{1}{N} \sum_{k=1}^{N} v_{k} X_{k} \rightarrow \mathbb {E}X \int _{0}^{1} v(y) \,dy. $$

### Proof

This immediately follows by applying Theorem 1 to the i.i.d. sequence $Y_{k} = X_{k} - \mathbb {E}X$. It remains to show that *v̅* exists. Denoting $\overline{v}_{N} = \frac{1}{N} \sum_{k=1}^{N} v_{k}$ as the sample mean among the first *N* coefficients with $|v_{k}| \leq M$ for all *k*, we show that $(\overline{v}_{N})_{N \geq 1}$ is a Cauchy sequence. We have

65 $$ \begin{aligned}[b] \vert \overline{v}_{N} - \overline{v}_{N+1} \vert &= \Biggl\vert \frac{1}{N} \sum _{k=1}^{N} v_{k} - \frac{1}{N+1} \sum_{k=1}^{N+1} v_{k} \Biggr\vert \\ &= \frac{1}{N(N+1)} \Biggl\vert (N+1) \sum_{k=1}^{N} v_{k} - N \sum_{k=1}^{N+1} v_{k} \Biggr\vert \\ &\leq \frac{2M}{N+1} < \epsilon \end{aligned} $$

for sufficiently large *N*. Hence, the limit $\overline{v} = \lim_{N \rightarrow \infty } \overline{v}_{N}$ exists. In the case where $v_{i} = v(i/N)$, it is clear that $\overline{v} = \int _{0}^{1} v(x) \,dx$ by taking Riemann sums. □

We applied Corollary 1 to random sequences of the form $X_{k} = f(\Delta _{k}, \tau ^{0})$ where $\Delta _{k}$ is Gaussian distributed, and $\tau ^{0}$ is the constant baseline lag. The eigenfunction coefficients $v_{k}$ are from ansatz $\epsilon _{k}(t) = v_{k} e^{\lambda t}$ as $N \rightarrow \infty $.

## Appendix C: Numerical setup

All numerical simulations in Sects. 6, 7 were done using MATLAB’s ddesd function. We have a system of delay differential equations for the phases $\theta _{i}(t)$, $i \leq N$ given by Eq. (1) and an $N^{2}$ system of ordinary differential equations for the state-dependent delays $\tau _{ij}(t)$, $1 \leq i$, $j \leq N$ given by Eq. (3). The initial data before starting time $t = 0$ are as follows. We have a history function $\varphi : [-r, 0] \rightarrow \mathbb {R}^{N}$ for the phases, such that $\theta _{i}(t) = \varphi _{i}(t)$ for all $t \leq 0$. We considered only the simple case where delays $\tau _{ij}(t)$ have a unique baseline lag $\tau _{ij}(0) = \tau ^{0} \in \mathbb {R}$. As shown in Appendix A, it is sufficient to consider $C^{1}$ initial phase functions $\varphi (t)$ to ensure unique solutions. However, we are not guaranteed to have reasonably behaving solutions unless we have continuity of $\theta '(t)$ at $t = 0$ [19]. This is also a necessary condition for providing accurate numerical simulations, as interpolation steps in ddesd rely on $\theta '(t)$ being continuous everywhere.

For our numerical experiments in Sects. 6 and 7, we consider the linear initial functions $\varphi _{i}(t) = \varOmega _{0} t + \phi _{i}^{0}$, $i \leq N$ with respect to initial frequency $\varOmega _{0} \in \mathbb {R}$ and phase-offsets $(\phi _{1}^{0}, \ldots , \phi _{N}^{0}) \in \mathbb {R}^{N}$. As discussed above, we require that our linear initial function must satisfy the necessary condition Eq. (49). Given any initial $C^{1}$ function $\varphi (t)$ for $\theta (t)$, we define the modified $C^{1}$ function $\tilde{\varphi }(t)$ of $\varphi (t)$ that satisfies the necessary condition as follows. We can obtain the modified slope $\vec{v} \in \mathbb {R}^{N}$ by imposing the condition on $\varphi (t)$. That is,

66 $$ v_{i} = \frac{g}{N} \sum _{i=1}^{N} \sin \bigl(\varphi _{j} \bigl(-\tau ^{0}\bigr) - \varphi _{i}(0)\bigr). $$

Then for each *i* we define the cubic polynomial $p_{i}: \mathbb {R}\rightarrow \mathbb {R}$ that interpolates $\varphi (t)$ between $t = 0, -\tau ^{0}$ using the modified slope. That is, $p_{i}(t) = \varphi _{i}(t)$ at $t = 0, -\tau ^{0}$ and $p_{i}'(0) = v_{i}$, $p_{i}'(-\tau ^{0}) = \varphi _{i}(-\tau ^{0})$. Then the modified initial function $\tilde{\varphi }(t)$ is given by

67 $$ \tilde{\varphi }_{i}(t) = \textstyle\begin{cases} \varphi _{i}(t), & t < -\tau ^{0}, \\ p_{i}(t), & -\tau ^{0} \leq t \leq 0, \end{cases} $$

for $i \leq N$. All numerical trials were conducted using the corresponding modified functions $\tilde{\varphi }_{i}(t)$ of $\varphi _{i}(t) = \varOmega t + \phi _{i}^{0}$, $i \leq N$.

## Footnotes

1 We can represent any connection topology of the network as an integral operator $a:[0,1]^{2} \rightarrow \{0,1\}$ such that $a_{ij} = a(i/N, j/N)$, for which we find the eigenfunction $v:[0,1] \rightarrow \mathbb {C}$ satisfying

$$ \sigma v(x) = \int _{0}^{1} a(x,y)v(y) \,dy $$

with respect to eigenvalue *σ*.

2 Trials were done using other types of initial functions $\varphi _{i}(t)$, and yielded similar convergence results. However, the synchronization occurred at a slower rate.

## Abbreviations

i.i.d.: independent and identically distributed
